# Supplementary material for: NRF2 modulates WNT signaling pathway to enhance photodynamic therapy resistance in oral leukoplakia
Source: EMBO Mol Med. 2025 Jun 10;17(7):1794–824. doi: 10.1038/s44321-025-00256-w (PMC12254380; doi:10.1038/s44321-025-00256-w)
Supplement: Supplementary file 1 — Appendix [file 44321_2025_256_MOESM1_ESM.pdf]

Appendix Table of Contents

Appendix Table S1	P1-2
Appendix Table S2	P3
Appendix Table S3	P4
Appendix Table S4	P5
Appendix Table S5	P6
Appendix Table S6	P7
Appendix Table S7	P8
Appendix Table S8	P9-10

Appendix Table S1

Figure panel	Comparison	Statistical test	p-value	Sample size (n)					
				Basal_Sensitive	Basal_Resistant	Cycling_Sensitive	Cycling_Resistant	Differentiated_Sensitive	Differentiated_Resistant
**Figure 2B**	Differentiated_Sensitive vs. Differentiated_Resistant	Wilcoxon rank-sum test	p<2.2e-16	1466	799	1042	330	820	474
	Cycling_Sensitive vs. Cycling_Resistant		p<2.2e-16						
	Basal_Sensitive vs. Basal_Resistant		p<2.2e-16						
				Basal_Sensitive	Basal_Resistant	Cycling_Sensitive	Cycling_Resistant	Differentiated_Sensitive	Differentiated_Resistant
**Figure 2D**	(NRF2-ARE) Basal_Sensitive vs. Basal_Resistant	Wilcoxon rank-sum test	p<2.2e-16	1466	799	1042	330	820	474
	(NRF2-ARE) Cycling_Sensitive vs. Cycling_Resistant		p<2.2e-16						
	(NRF2-ARE) Differentiated_Sensitive vs. Differentiated_Resistant		p<2.2e-16						
	(Keratinization) Basal_Sensitive vs. Basal_Resistant		p<2.2e-16						
	(Keratinization) Cycling_Sensitive vs. Cycling_Resistant		p<2.2e-16						
	(Keratinization) Differentiated_Sensitive vs. Differentiated_Resistant		p=0.0016						
				sensitive	resistant				
**Figure 2F**	sensitive vs. resistant	Student's *t*-test	p=0.0147	11	9				
				sensitive	resistant				
**Figure 2G**	sensitive vs. resistant	Student's *t*-test	p<0.0001	48	20				
				sensitive	resistant				
**Figure 2H**	sensitive vs. resistant	ROC analysis (Wilcoxon rank-sum test)	p<0.0001	48	20				
				sensitive	resistant				
**Figure 2I**	sensitive vs. resistant	Student's *t*-test	p=0.0002	18	11				
				sensitive	resistant				
**Figure 2J**	sensitive vs. resistant	ROC analysis(Wilcoxon rank-sum test)	p=0.001	18	11				
				Total					
**Figure 2K**	K.I.$_1$	Student's *t*-test for Pearson correlation coefficient	p=0.0011	20					
	K.I.$_2$		p<0.0001	20					
				DOK	DOK-RP				
**Figure 3B**	DOK vs. DOK-RP (3 J/cm2)	Student's t-test	p=0.0009	4	4				
	DOK vs. DOK-RP (6 J/cm2)		p=0.025	4	4				
	DOK vs. DOK-RP (9 J/cm2)		p=0.0381	4	4				
	DOK vs. DOK-RP (12 J/cm2)		p=0.0798	4	4				
				DOK	DOK-RP				
**Figure 3D**	DOK vs. DOK-RP	Student's *t*-test	p=0.02	3	3				
				DOK	DOK-RP				
**Figure 3E**	DOK vs. DOK-RP	Student's *t*-test	p=0.0045	6	6				
				DOK	DOK-RP				
**Figure 3F**	DOK vs. DOK-RP	Student's *t*-test	p=0.0033	3	3				
				Control	OE-NRF2				
**Figure 4B**	Control vs. OE-NRF2 (3 J/cm2)	Student's *t*-test	p=0.0004	4	4				
	Control vs. OE-NRF2 (6 J/cm2)		p<0.0001	4	4				
	Control vs. OE-NRF2 (9 J/cm2)		p=0.0005	4	4				
	Control vs. OE-NRF2 (12 J/cm2)		p<0.0001	4	4				
				DMSO(DOK)	Brusatol(DOK)				
**Figure 4D**	(DOK) DMSO vs. Brusatol (3 J/cm2)	Student's *t*-test	p<0.0001	4	4				
	(DOK) DMSO vs. Brusatol (6 J/cm2)		p<0.0001	4	4				
	(DOK) DMSO vs. Brusatol (9 J/cm2)		p=0.0001	4	4				
	(DOK) DMSO vs. Brusatol 2 (12 J/cm2)		p=0.0005	4	4				
				DMSO(DOK-RP)	Brusatol(DOK-RP)				
**Figure 4E**	(DOK-RP) DMSO vs. Brusatol (3 J/cm2)	Student's *t*-test	p<0.0001	4	4				
	(DOK-RP) DMSO vs. Brusatol (6 J/cm2)		p<0.0001	4	4				
	(DOK-RP) DMSO vs. Brusatol (9 J/cm2)		p<0.0001	4	4				
	(DOK-RP) DMSO vs. Brusatol 2 (12 J/cm2)		0.0023	4	4				
				Control	OE-NRF2				
**Figure 4F**	Control vs. OE-NRF2	Student's *t*-test	p=0.002	3	3				
				DMSO(DOK)	Brusatol(DOK)				
	(DOK) DMSO vs. Brusatol	Student's *t*-test	p=0.0168	4	4				
				DMSO(DOK-RP)	Brusatol(DOK-RP)				
	(DOK-RP) DMSO vs. Brusatol	Student's *t*-test	p=0.0031	3	3				
				Control	OE-NRF2				
**Figure 4G**	Control vs. OE-NRF2	Student's *t*-test	p=0.0004	3	3				
				DMSO(DOK)	Brusatol(DOK)				
	(DOK) DMSO vs. Brusatol	Student's *t*-test	p=0.0127	3	3				
				Basal_Sensitive	Basal_Resistant	Cycling_Sensitive	Cycling_Resistant	Differentiated_Sensitive	Differentiated_Resistant
**Figure 5A**	(WNT) Basal_Sensitive vs. Basal_Resistant	Wilcoxon rank-sum test	p<2.2e-16	1466	799	1042	330	820	474
	(WNT) Cycling_Sensitive vs. Cycling_Resistant		p<2.2e-16						
	(WNT) Differentiated_Sensitive vs. Differentiated_Resistant		p<2.2e-16						
				sensitive	resistant				
**Figure 5B**	sensitive vs. resistant	Student's *t*-test	p=0.0005	9	8				
				DMSO	Brusatol + BML284	Brusatol			
**Figure 5F**	DMSO vs. Brusatol + BML284 (3 J/cm2)	Student's *t*-test	p=0.0053	4	4	4			
	Brusatol vs. Brusatol + BML284 (3 J/cm2)		p=0.0052						
	DMSO vs. Brusatol + BML284 (6 J/cm2)		p=0.0003						
	Brusatol vs. Brusatol + BML284 (6 J/cm2)		p=0.0019						
	DMSO vs. Brusatol + BML284 (9 J/cm2)		p=0.0023						
	Brusatol vs. Brusatol + BML284 (9 J/cm2)		p=0.0625						
	DMSO vs. Brusatol + BML284 (12 J/cm2)		p=0.012						
	Brusatol vs. Brusatol + BML284 (12 J/cm2)		p=0.0617						
				DMSO	Brusatol + BML284	Brusatol			

**Figure 5G**	DMSO vs. Brusatol + BML284 (3 J/cm2)	Student's *t* -test	p=0.0182	4	4	4			
	Brusatol vs. Brusatol + BML284 (3 J/cm2)		p=0.0011						
	DMSO vs. Brusatol + BML284 (6 J/cm2)		p=0.0012						
	Brusatol vs. Brusatol + BML284 (6 J/cm2)		p=0.0001						
	DMSO vs. Brusatol + BML284 (9 J/cm2)		p=0.0041						
	Brusatol vs. Brusatol + BML284 (9 J/cm2)		p=0.0228						
	DMSO vs. Brusatol + BML284 (12 J/cm2)		p=0.005						
	Brusatol vs. Brusatol + BML284 (12 J/cm2)		p=0.0048						
				Control	OE-NRF2 +IWR1	OE-NRF2			
**Figure 5H**	Control vs. OE-NRF2 +IWR1 (3 J/cm2)	Student's *t* -test	p=0.4085	4	4	4			
	OE-NRF2 +IWR1 vs. OE-NRF2 (3 J/cm2)		p=0.0085						
	Control vs. OE-NRF2 +IWR1 (6 J/cm2)		p=0.0045						
	OE-NRF2 +IWR1 vs. OE-NRF2 (6 J/cm2)		p=0.0059						
	Control vs. OE-NRF2 +IWR1 (9 J/cm2)		p=0.0099						
	OE-NRF2 +IWR1 vs. OE-NRF2 (9 J/cm2)		p=0.1898						
	Control vs. OE-NRF2 +IWR1 (12 J/cm2)		p=0.0005						
	OE-NRF2 +IWR1 vs. OE-NRF2 (12 J/cm2)		p=0.0016						
				DOK	DOK-RP				
**Figure 5K**	(IgG)DOK vs. DOK-RP	Student's *t* -test	p=0.8448	3	3				
	(CTNNB1-ARE)DOK vs. DOK-RP		p=0.0031	3	3				
				DOK	DOK-RP				
**Figure 5L**	DOK vs. DOK-RP	Student's *t* -test	p<0.0001	3	3				
				NC	Brusatol	PDT	Combo		
**Figure 6D**	NC vs. PDT	Student's *t* -test	p=0.0274	4	4	4	4		
	NC vs. Combo		p <0.0001						
	Brusatol vs. Combo		p=0.0126						
	PDT vs. Combo		p=0.0386						
				NC	Brusatol	PDT	Combo		
**Figure 6G**	NC vs. Combo	Student's *t* -test	p=0.0013	4	4	4	4		
	Brusatol vs. Combo		p=0.0086						
	PDT vs. Combo		p=0.0484						
				NC	Brusatol	PDT	Combo		
**Figure 6I**	NC vs. PDT	Student's *t* -test	p=0.0086	4	4	4	4		
	NC vs. Combo		p=0.0007						
	Brusatol vs. Combo		p<0.0001						
	PDT vs. Combo		p=0.0029						
				sensitive	resistant				
**Figure EV2B**	sensitive vs. resistant	Student's *t* -test	p=0.02	11	9				
				sensitive	resistant				
**Figure EV2C**	(Discovery Cohort)sensitive vs. resistant	Student's *t* -test	p=0.0025	11	9				
	(Validation Cohort -Internal) sensitive vs. resistant		p=0.0046	48	20				
	(Validation Cohort - External)sensitive vs. resistant		p<0.0001	18	11				
				Total					
**Figure EV2D**	K.I.$_1$	Student's t-test for Pearson correlation coefficient	p<0.0001	68					
	K.I.$_2$		p=0.0001	68					
				DOK	DOK-RP				
**Figure EV3B**	DOK vs. DOK-RP	Student's *t* -test	p=0.014	5	5				
				DOK	DOK-RP				
**Figure EV3C**	HMOX1 (DOK vs. DOK-RP)	Student's *t* -test	p<0.0001	3	3				
	SLC7A11(DOK vs. DOK-RP)		p=0.003	3	3				
	SOD1(DOK vs. DOK-RP)		p=0.001	3	3				
	FTH1 (DOK vs. DOK-RP)		p=0.06	3	3				
	GCLC (DOK vs. DOK-RP)		p=0.05	3	3				
	GCLM (DOK vs. DOK-RP)		p=0.001	3	3				
	GGT1(DOK vs. DOK-RP)		p<0.0001	3	3				
				DOK	DOK-RP				
**Figure EV3D**	DOK vs. DOK-RP	Student's *t* -test	p=0.0034	3	3				
				Control	OE-NRF2				
**Figure EV4A**	Control vs. OE-NRF2	Student's *t* -test	p<0.0001	3	3				
				Total					
**Figure EV5F**	K.I.$_1$	Student's *t* -test for Pearson correlation coefficient	p=0.006	17					
	K.I.$_2$		p=0.004	17					
				DMSO	Brusatol + BML284	Brusatol			
**Figure EV5G**	(DOK) DMSO vs. Brusatol + BML284	Student's *t* -test	p=0.0003	3	3	3			
	(DOK) Brusatol + BML284 vs. Brusatol		p=0.0001						
				DMSO	Brusatol + BML284	Brusatol			
**Figure EV5H**	(DOK-RP) DMSO vs. Brusatol + BML284	Student's *t* -test	p=0.0001	3	3	3			
	(DOK-RP) Brusatol + BML284 vs. Brusatol	Student's *t* -test	p<0.0001						
				Control	OE-NRF2 +IWR1	OE-NRF2			
**Figure EV5I**	Control vs. OE-NRF2 +IWR1	Student's *t* -test	p<0.0001	3	3	3			
	OE-NRF2 +IWR1 vs. OE-NRF2		p=0.0292						
				NC	Brusatol				
**Figure EV6B**	(NRF2) NC vs. Brusatol	Student's *t* -test	p=0.0006	3	3				
	(p-NRF2) NC vs. Brusatol		p=0.0059						
	(CATENIN) NC vs. Brusatol		p=0.0318						

Appendix Table S2 Pathway enrichment analysis of NRF2 downstream genes.

Description	p value	p.adjust	geneID
cell redox homeostasis	2.08E-05	1.62E-02	TXN/NFE2L2/NQO1/PRDX1
cell-cell junction organization	2.26E-05	1.62E-02	DSG1/TJP1/CDH1/GJA1/PERP/JUP/PKP1
cell-cell junction assembly	3.78E-05	1.66E-02	DSG1/TJP1/CDH1/GJA1/JUP/PKP1
cellular oxidant detoxification	4.63E-05	1.66E-02	GPX2/TXN/NFE2L2/NQO1/PRDX1
response to toxic substance	7.40E-05	2.04E-02	GPX2/SDC1/TXN/CDH1/NFE2L2/NQO1/PRDX1
removal of superoxide radicals	1.01E-04	2.04E-02	NFE2L2/NQO1/PRDX1
cellular detoxification	1.11E-04	2.04E-02	GPX2/TXN/NFE2L2/NQO1/PRDX1
cellular response to oxygen radical	1.30E-04	2.04E-02	NFE2L2/NQO1/PRDX1
cellular response to superoxide	1.30E-04	2.04E-02	NFE2L2/NQO1/PRDX1
cellular response to toxic substance	1.57E-04	2.04E-02	GPX2/TXN/NFE2L2/NQO1/PRDX1
response to superoxide	1.84E-04	2.05E-02	NFE2L2/NQO1/PRDX1
response to oxidative stress	1.86E-04	2.05E-02	G6PD/GPX2/SDC1/TXN/DHCR24/NFE2L2/NQO1/PRDX1
response to oxygen radical	2.05E-04	2.10E-02	NFE2L2/NQO1/PRDX1
regulation of peptidase activity	2.40E-04	2.29E-02	CTSD/SERPINB5/DHCR24/NRDC/CAST/PERP/SFN
establishment of skin barrier	3.02E-04	2.71E-02	KRT16/SFN/TMEM79
keratinization	3.34E-04	2.82E-02	KRT16/SFN/KRT6A/TMEM79
cell junction assembly	3.86E-04	3.08E-02	DSG1/EIF4G1/TJP1/CDH1/GJA1/MYCBP2/JUP/PKP1
detoxification	4.16E-04	3.14E-02	GPX2/TXN/NFE2L2/NQO1/PRDX1

Appendix Table S3. Clinical and pathological information of patients undergoing scRNA-seq.

NO.	Groups	Gender	Age	Dysplasia
OLK51	Sensitive	M	46	None
OLK52	Sensitive	M	53	Moderate
OLK55	Sensitive	M	30	None
OLK57	Resistant	M	50	Mild
OLK59	Resistant	F	52	None
OLK60	Resistant	F	45	Mild

Appendix Table S4. Clinical and pathological information of patients in the discovery cohort.				
**NO.**	**Groups**	**Gender**	**Age**	**Dysplasia**
1	Resistant	M	44	Mild
2	Resistant	M	44	Moderate
3	Resistant	M	52	Mild
4	Resistant	F	75	Severe
5	Resistant	M	44	Mild
6	Resistant	M	66	Moderate
7	Resistant	F	49	Mild
8	Resistant	F	70	Severe
9	Resistant	M	28	None
10	Sensitive	F	54	Severe
11	Sensitive	M	30	Mild
12	Sensitive	M	68	Severe
13	Sensitive	F	65	Mild
14	Sensitive	F	71	Mild
15	Sensitive	M	67	Severe
16	Sensitive	F	54	Moderate
17	Sensitive	M	32	Mild
18	Sensitive	M	41	None
19	Sensitive	M	71	None
20	Sensitive	F	63	Severe

Appendix Table S5. Clinical and pathological information of patients in internal validation cohort.

NO.	Groups	Gender	Age	Dysplasia
1	Resistant	F	38	None
2	Resistant	F	39	None
3	Resistant	M	39	Moderate
4	Resistant	F	43	None
5	Resistant	M	44	Mild
6	Resistant	M	45	None
7	Resistant	F	47	Severe
8	Resistant	M	50	Severe
9	Resistant	F	51	Mild
10	Resistant	F	52	None
11	Resistant	F	53	Moderate
12	Resistant	F	53	Severe
13	Resistant	F	55	Severe
14	Resistant	M	59	Mild
15	Resistant	F	62	Moderate
16	Resistant	M	62	Severe
17	Resistant	F	64	Severe
18	Resistant	F	66	None
19	Resistant	F	66	Mild
20	Resistant	F	69	Moderate
21	Sensitive	F	24	None
22	Sensitive	M	33	Mild
23	Sensitive	F	33	Moderate
24	Sensitive	M	35	Severe
25	Sensitive	F	37	Severe
26	Sensitive	M	38	Mild
27	Sensitive	F	41	None
28	Sensitive	M	42	None
29	Sensitive	M	43	None
30	Sensitive	F	45	Moderate
31	Sensitive	M	46	Mild
32	Sensitive	F	47	None
33	Sensitive	F	48	Severe
34	Sensitive	M	49	Moderate
35	Sensitive	F	50	Severe
36	Sensitive	F	50	Moderate
37	Sensitive	M	51	Moderate
38	Sensitive	M	51	None
39	Sensitive	M	52	None
40	Sensitive	F	53	Moderate
41	Sensitive	F	54	Moderate
42	Sensitive	F	56	Mild
43	Sensitive	F	56	Severe
44	Sensitive	F	57	Mild
45	Sensitive	F	58	None
46	Sensitive	M	59	None
47	Sensitive	F	60	Moderate
48	Sensitive	F	60	Moderate
49	Sensitive	M	61	None
50	Sensitive	F	62	Moderate
51	Sensitive	M	63	None
52	Sensitive	F	63	Moderate
53	Sensitive	F	63	Moderate
54	Sensitive	F	63	Moderate
55	Sensitive	F	64	Mild
56	Sensitive	M	64	Moderate
57	Sensitive	F	65	Severe
58	Sensitive	M	66	Mild
59	Sensitive	F	66	Mild
60	Sensitive	F	66	Moderate
61	Sensitive	F	69	Moderate
62	Sensitive	F	70	Mild
63	Sensitive	F	70	Moderate
64	Sensitive	F	71	Moderate
65	Sensitive	M	72	Moderate
66	Sensitive	F	75	Severe
67	Sensitive	F	77	None
68	Sensitive	F	79	Mild

Appendix Table S6. Clinical and pathological information of patients in external validation cohort.

1	Sensitive	F	58	Mild
2	Resistant	F	81	Severe
3	Resistant	F	48	Mild
4	Sensitive	M	55	Severe
5	Sensitive	M	64	Mild
6	Sensitive	M	67	Moderate
7	Sensitive	M	36	Severe
8	Resistant	F	63	Moderate
9	Resistant	F	37	Mild
10	Resistant	F	78	None
11	Sensitive	M	60	Severe
12	Resistant	F	64	Severe
13	Sensitive	M	42	None
14	Resistant	M	65	Severe
15	Sensitive	M	57	Moderate
16	Sensitive	M	34	None
17	Sensitive	M	43	Moderate
18	Sensitive	M	35	Mild
19	Sensitive	M	63	Severe
20	Sensitive	F	52	Mild
21	Sensitive	M	64	Mild
22	Resistant	M	53	Severe
23	Sensitive	M	65	Mild
24	Resistant	M	38	Mild
25	Resistant	F	59	Severe
26	Sensitive	F	40	Severe
27	Sensitive	F	59	Mild
28	Sensitive	F	57	Mild
29	Resistant	M	57	None

Appendix Table S7. PCR primers.			
**Application**	**Genes**	**Primers**	
RT-qPCR	*GAPDH*	F	GGAGCGAGATCCCTCCAAAAT
		R	GGCTGTTGTCATACTTCTCATGG
	HMOX1	F	AAGACTGCGTTCCTGCTCAAC
		R	AAAGCCCTACAGCAACTGTCG
	SLC7A11	F	TTTTCTGAGCGGCTACTGGG
		R	CAGCTGGTAGAGGAGTGTGC
	SOD1	F	GGTGGGCCAAAGGATGAAGAG
		R	CCACAAGCCAAACGACTTCC
	FTH1	F	CCCCCATTTGTGTGACTTCAT
		R	GCCCGAGGCTTAGCTTTCATT
	GCLC	F	TCCAGGTGACATTCCAAGCC
		R	GAAATCACTCCCCAGCGACA
	GCLM	F	CTCCTGCTGTGTGATGCCA
		R	CTCGTGCGCTTGAATGTCAG
	GGT1	F	CTGGGGAGATCCGAGGCTAT
		R	GATGACGGTCCGCTTGTTTTC
	NRF2	F	TCAGCGACGGAAAGAGTATGA
		R	CCACTGGTTTCTGACTGGATGT
CUT&RUN	*CTNNB1-ARE*	F	CCTAGTGACAAGTGGAACCAGA
		R	CCTAGTGACAAGTGGAACCAGAT

Appendix Table S8 Antibodies and Dilution Information			
Antibodies			Dilutions
HMOX1	IB	Abcam	1:1000
NRF2	IB/IHC	Abcam	1:1000 (IB); 1:500 (IHC)
NRF2	CUT&RUN Assay	Proteintech	1:100
Phospho-Ser40-NRF2	IB/IHC/IF	Abcam	1:3000 (IB); 1:500 (IHC); 1:200 (IF)
SCL7A11	IB	Abcam	1:3000
Phospho-Ser9-GSK-3β	IB	Cell Signaling	1:1000
GSK-3β	IB	Abcam	1:2000
WNT3A	IB	Proteintech	1:500
WNT4	IB	Huabio	1:1000
WNT10A	IB	Huabio	1:1000
Cytokeratin 10	IB	Huabio	1:1000
Cytokeratin 1	IB	Huabio	1:1000
Loricrin	IB	Proteintech	1:1000
FZD6	IB	Cell Signaling	1:1000
Pan-Cytokeratin	IF	Abcam	1 μg/ml
KI67	IHC	Abcam	1:300
GAPDH	IB	Proteintech	1:20000
beta-catenin	IB/IHC	Proteintech	1:20000
Non-phospho (Active) β-Catenin (Ser45)	IB	CST	1:1000
α-tubulin	IB	Proteintech	1:20000
β-actin	IB/IHC	Proteintech	1:20000
Fuorochrome-conjugated secondary antibody	IF	ZSGB-BIO	1:10000
HRP-conjugated sencond antibody	IHC	Zsbio	1:10000
