# Supplementary figures and images for: NRF2 modulates WNT signaling pathway to enhance photodynamic therapy resistance in oral leukoplakia
Source: EMBO Mol Med. 2025 Jun 10;17(7):1794–824. doi: 10.1038/s44321-025-00256-w (PMC12254380; doi:10.1038/s44321-025-00256-w)

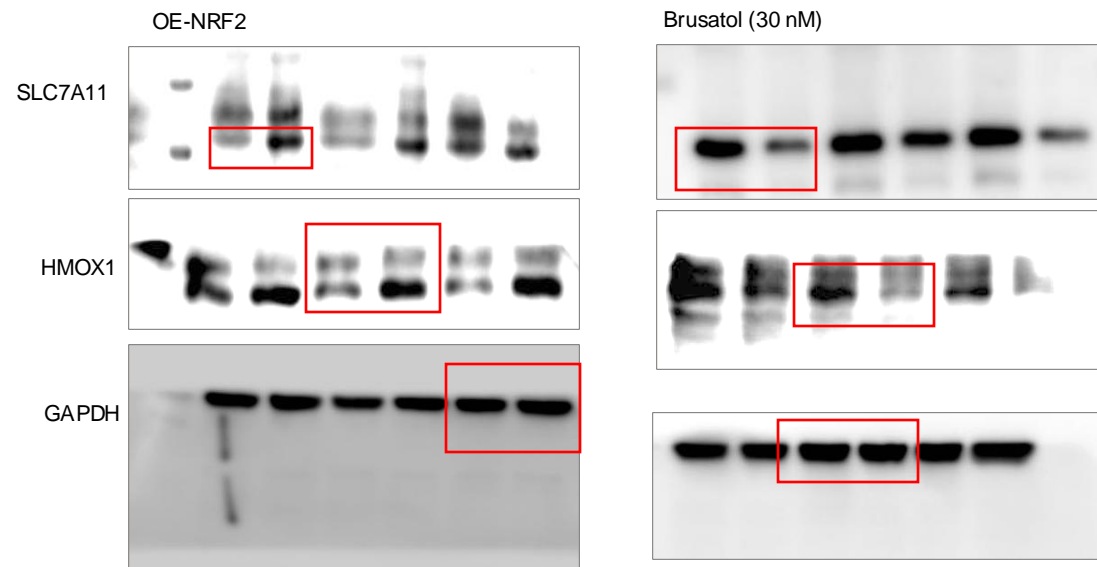

Supplement: Supplementary file 6 — Source data Fig. 4 [file 44321_2025_256_MOESM6_ESM.zip › Figure 4-source data/Figure 4H/Figure 4H.pdf]

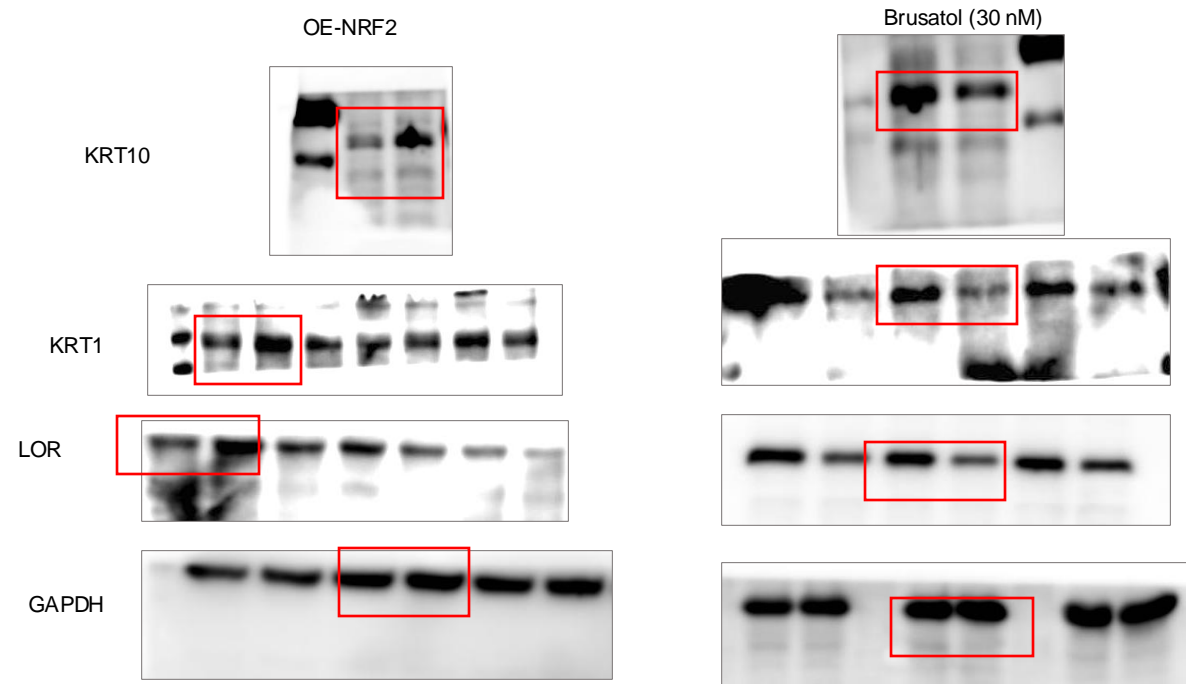

Supplement: Supplementary file 6 — Source data Fig. 4 [file 44321_2025_256_MOESM6_ESM.zip › Figure 4-source data/Figure 4I/figure 4I.pdf]

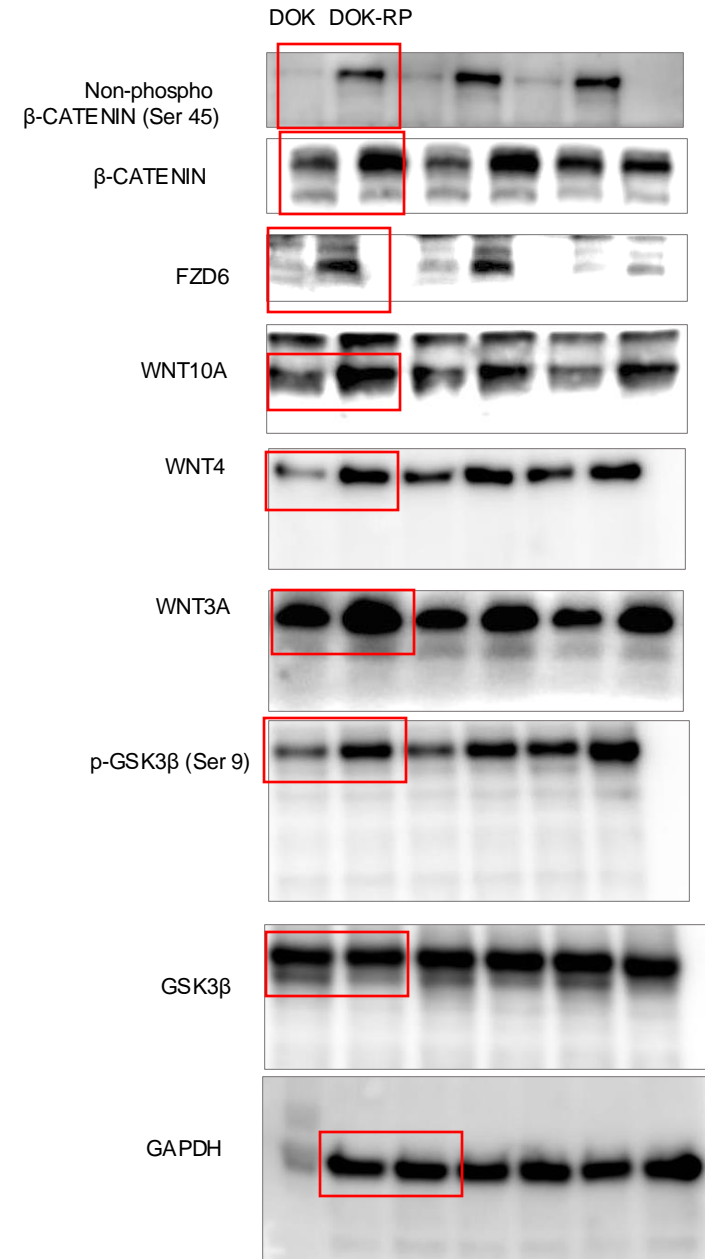

Supplement: Supplementary file 7 — Source data Fig. 5 [file 44321_2025_256_MOESM7_ESM.zip › Figure 5-source data/Figure 5C/figure 5C.pdf]

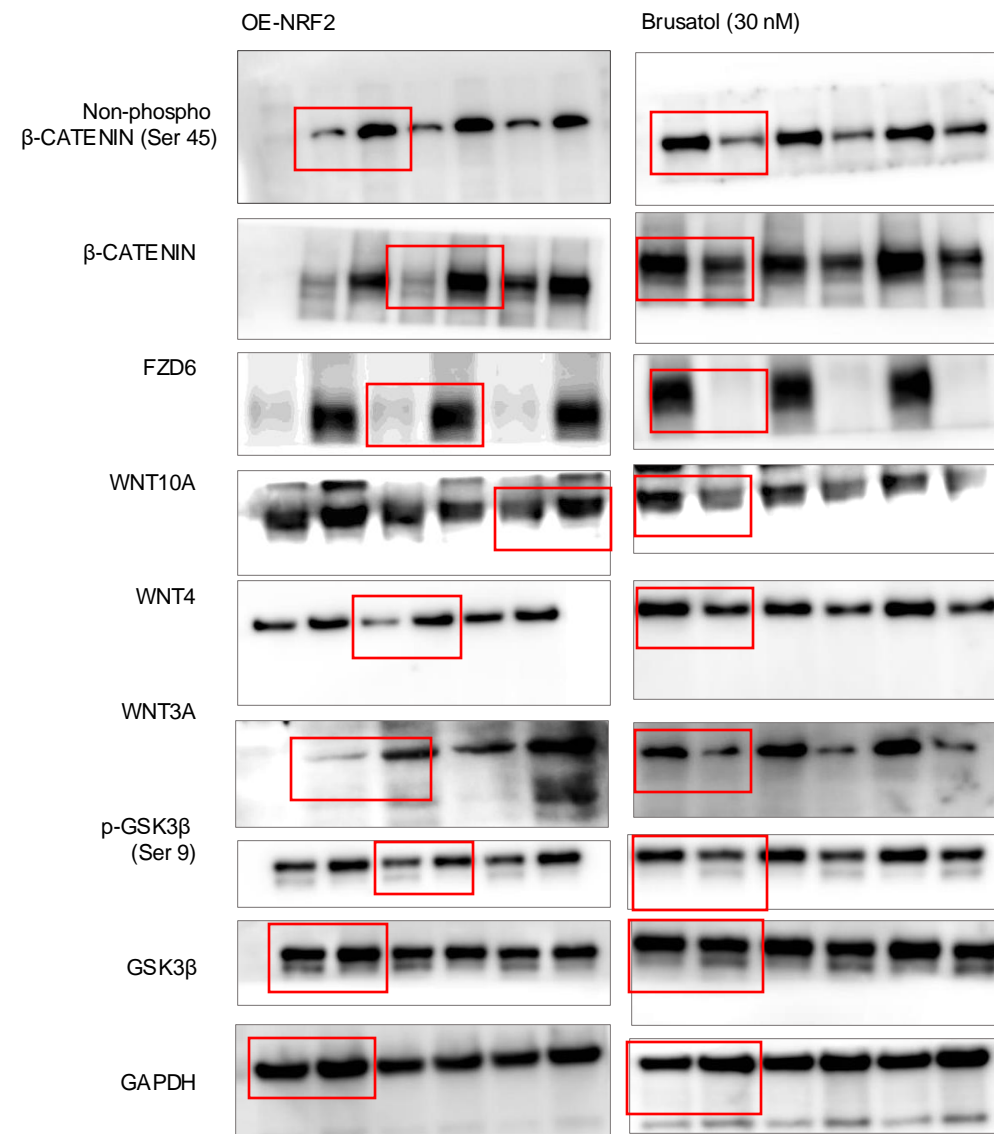

Supplement: Supplementary file 7 — Source data Fig. 5 [file 44321_2025_256_MOESM7_ESM.zip › Figure 5-source data/Figure 5D/5d.pdf]

BML284 (5  $\mu$ M)

IWR-1 (5  $\mu$ M)

KRT10

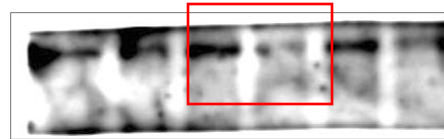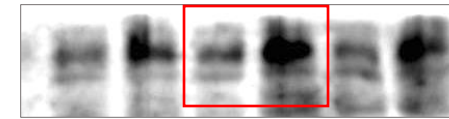

KRT1

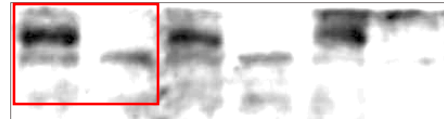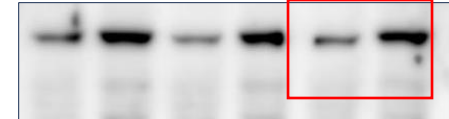

LOR

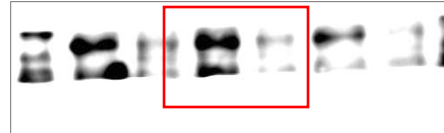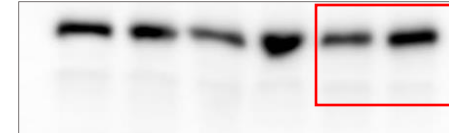

SLC7A11

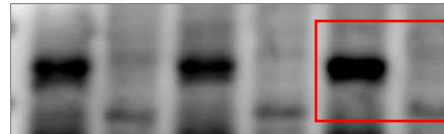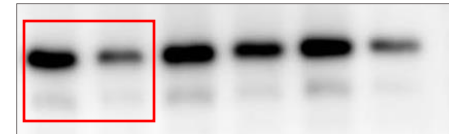

HMOX1

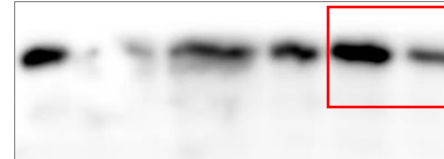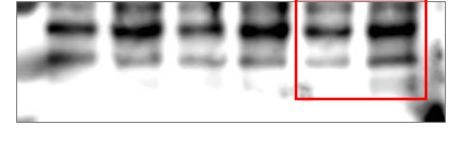

GAPDH

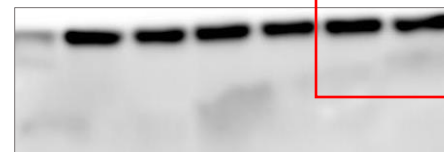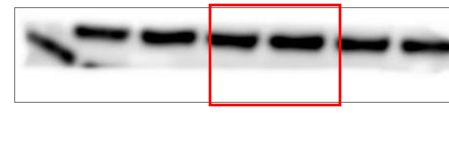

Supplement: Supplementary file 7 — Source data Fig. 5 [file 44321_2025_256_MOESM7_ESM.zip › Figure 5-source data/Figure 5E/5e.pdf]

resistant

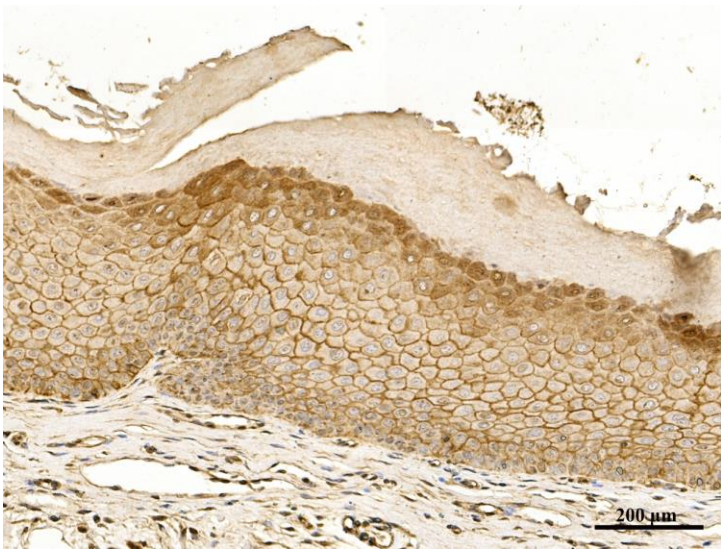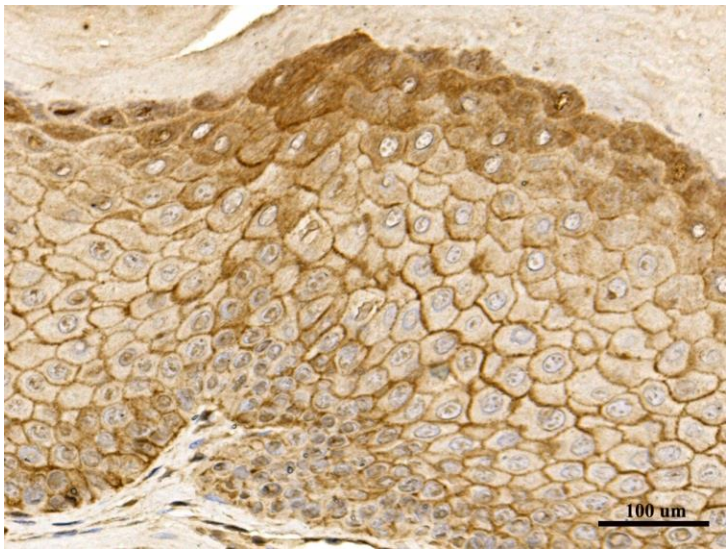

sensitive

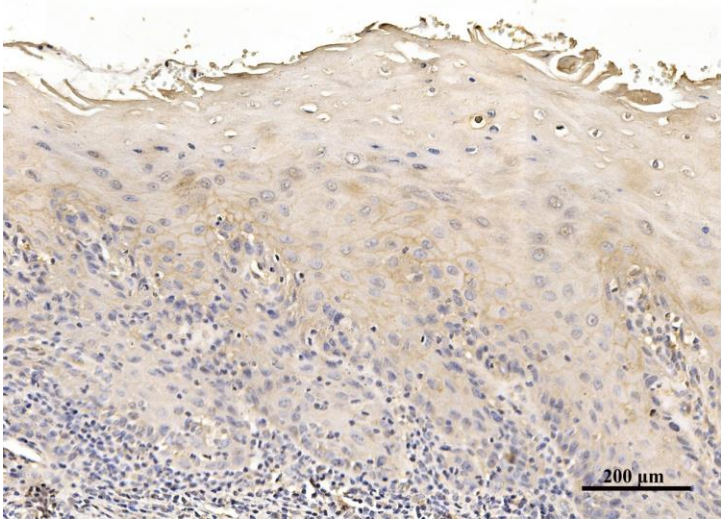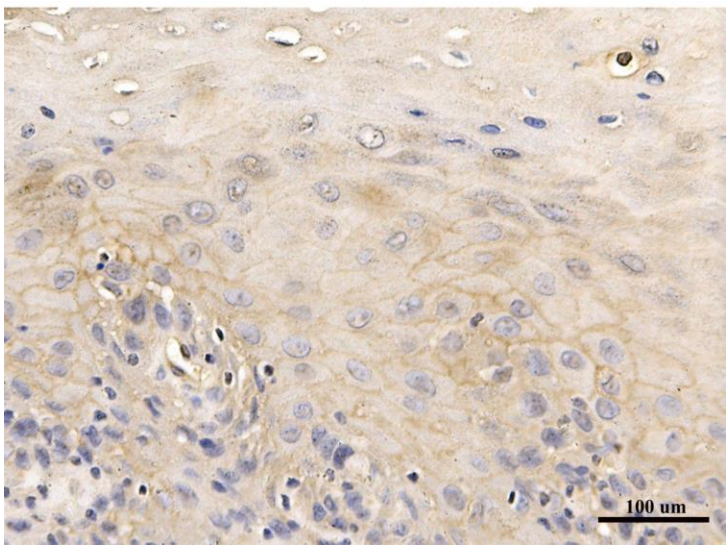

Supplement: Supplementary file 7 — Source data Fig. 5 [file 44321_2025_256_MOESM7_ESM.zip › Figure 5-source data/Figure 5B/figure 5b.pdf]

NC

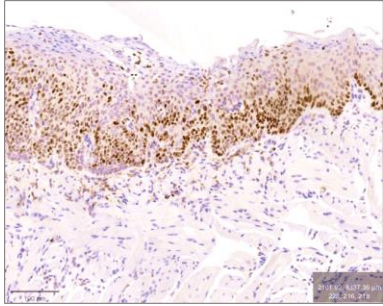

Brusatol

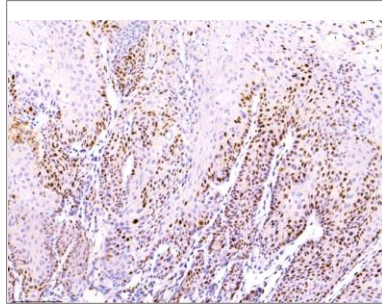

PDT

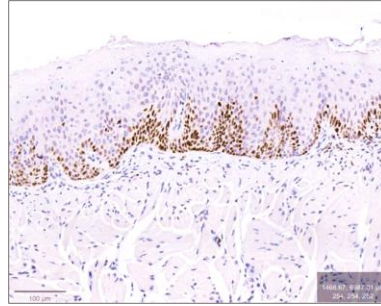

Combo

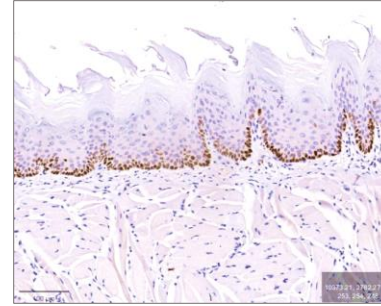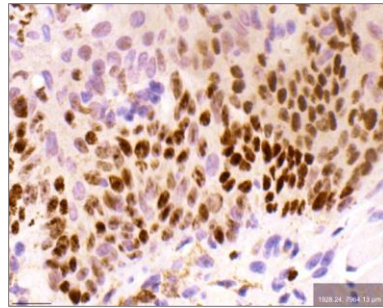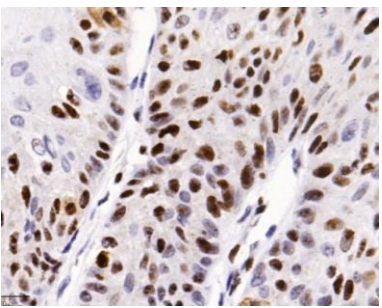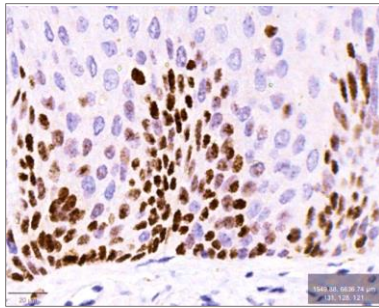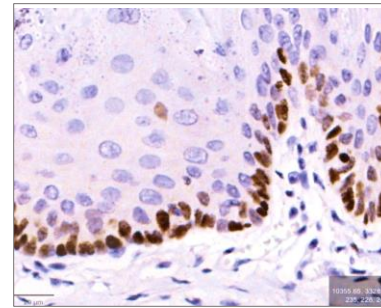

Supplement: Supplementary file 8 — Source data Fig. 6 [file 44321_2025_256_MOESM8_ESM.zip › Figure 6-source data/Figure 6H/figure 6H.pdf]

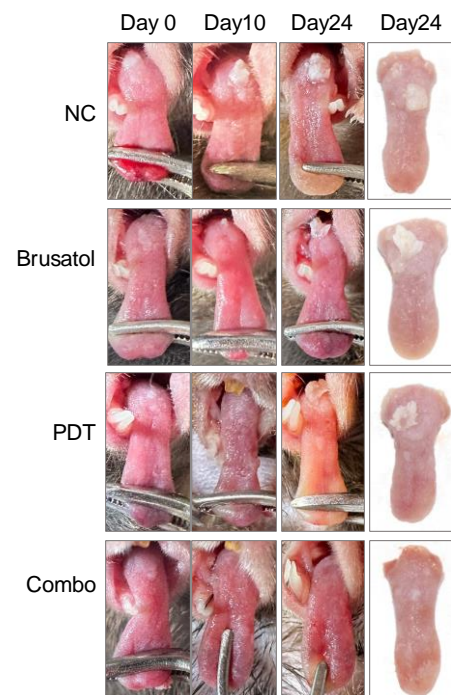

Supplement: Supplementary file 8 — Source data Fig. 6 [file 44321_2025_256_MOESM8_ESM.zip › Figure 6-source data/Figure 6B/μ╝öτñ║μûçτ¿┐8.pdf]

NC

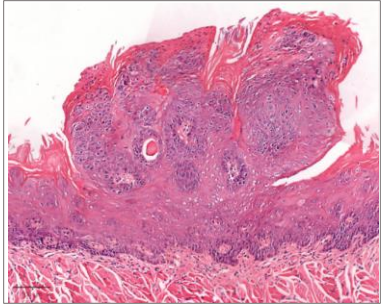

Brusatol

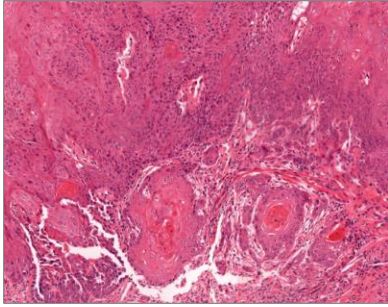

PDT

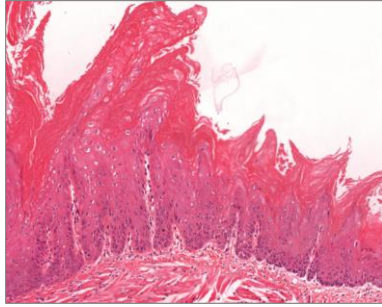

Combo

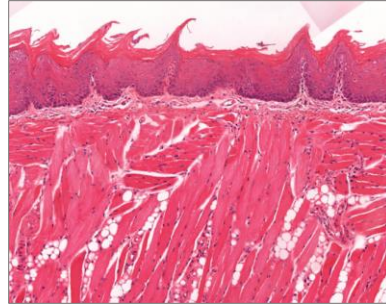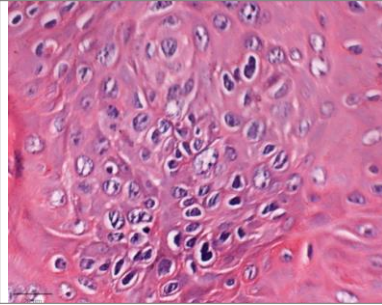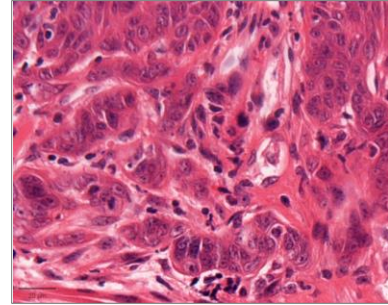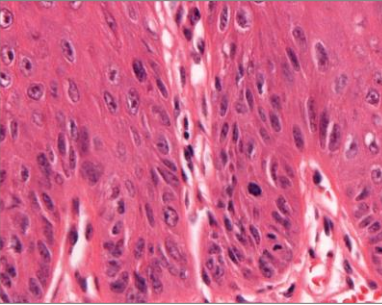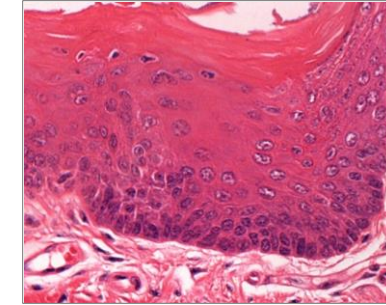

Supplement: Supplementary file 8 — Source data Fig. 6 [file 44321_2025_256_MOESM8_ESM.zip › Figure 6-source data/Figure 6E/figure 6E.pdf]

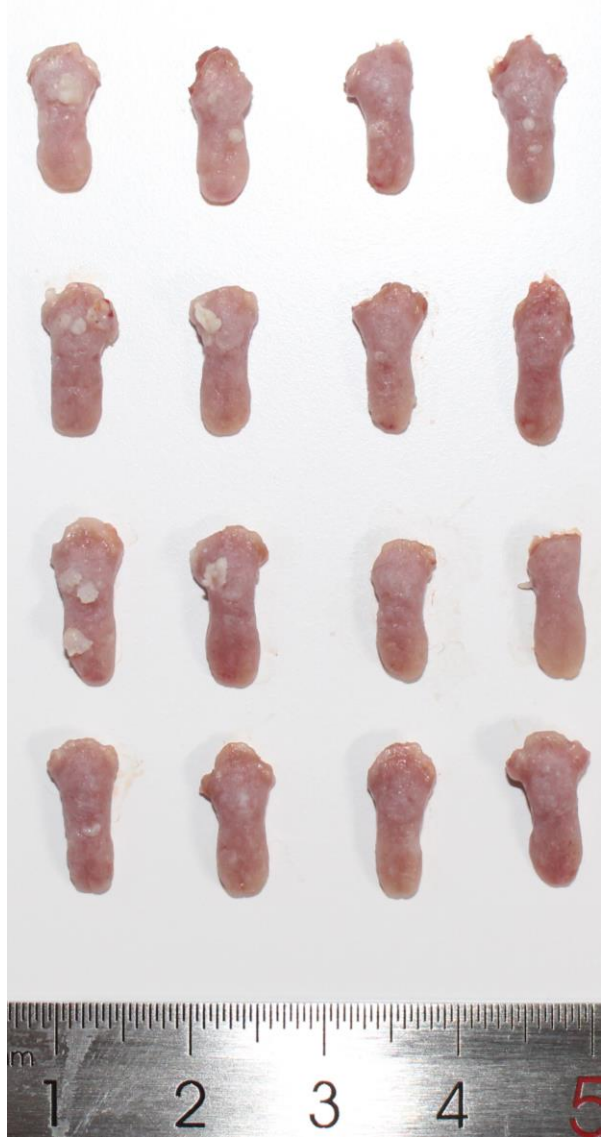

Supplement: Supplementary file 8 — Source data Fig. 6 [file 44321_2025_256_MOESM8_ESM.zip › Figure 6-source data/Figure 6C/Figure 6C.pdf]

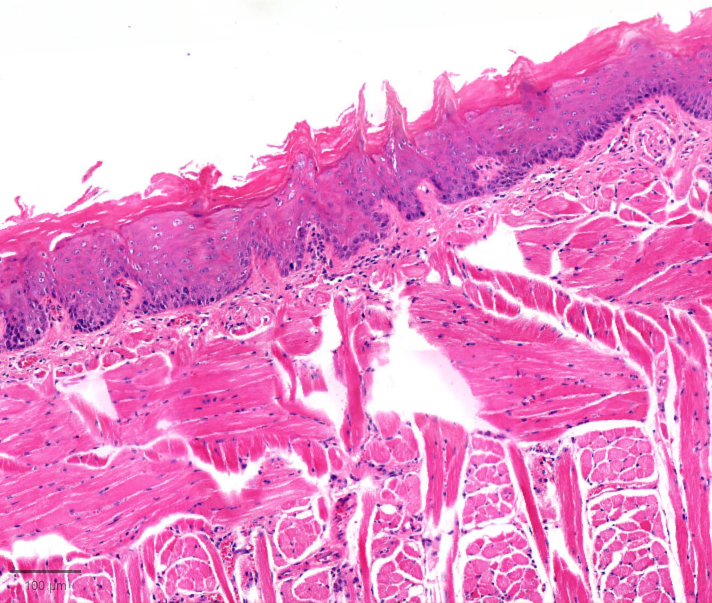

Supplement: Supplementary file 9 — Figure EV6 Source Data [file 44321_2025_256_MOESM9_ESM.zip › Figure EV6-source data/Figure EV6-A/(Revised version)EV6A-OLK-case2-5x.tif]

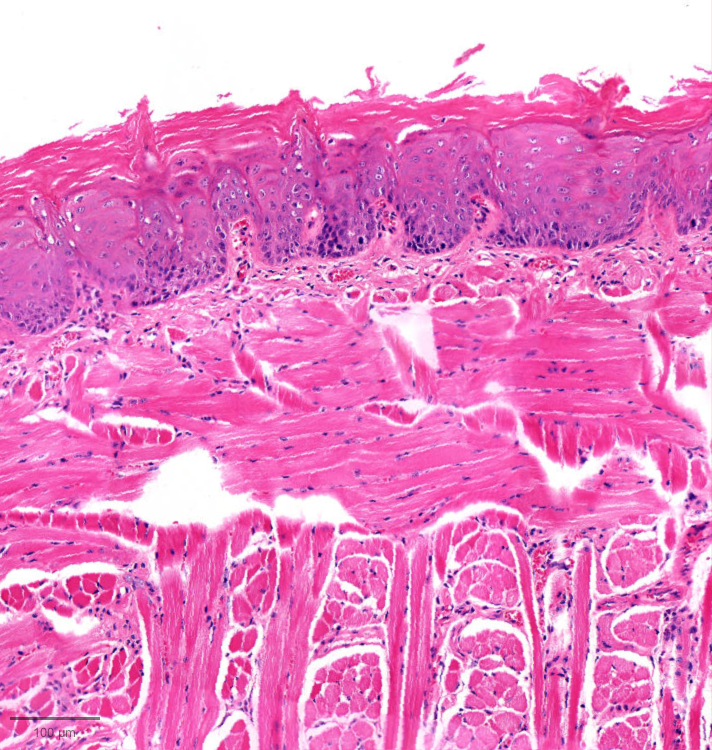

Supplement: Supplementary file 9 — Figure EV6 Source Data [file 44321_2025_256_MOESM9_ESM.zip › Figure EV6-source data/Figure EV6-A/(Revised version)EV6A-OLK-case2-10x.tif]

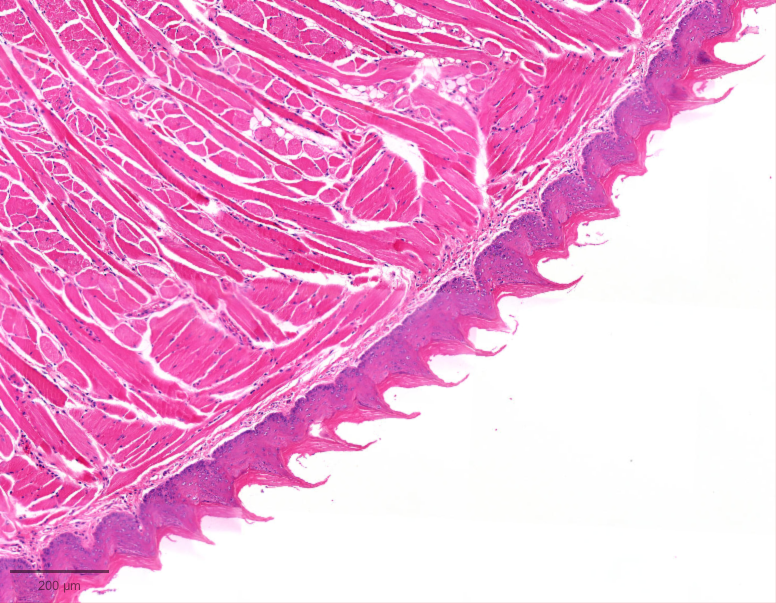

Supplement: Supplementary file 9 — Figure EV6 Source Data [file 44321_2025_256_MOESM9_ESM.zip › Figure EV6-source data/Figure EV6-A/(Revised version)EV6A-Normal-10x.tif]

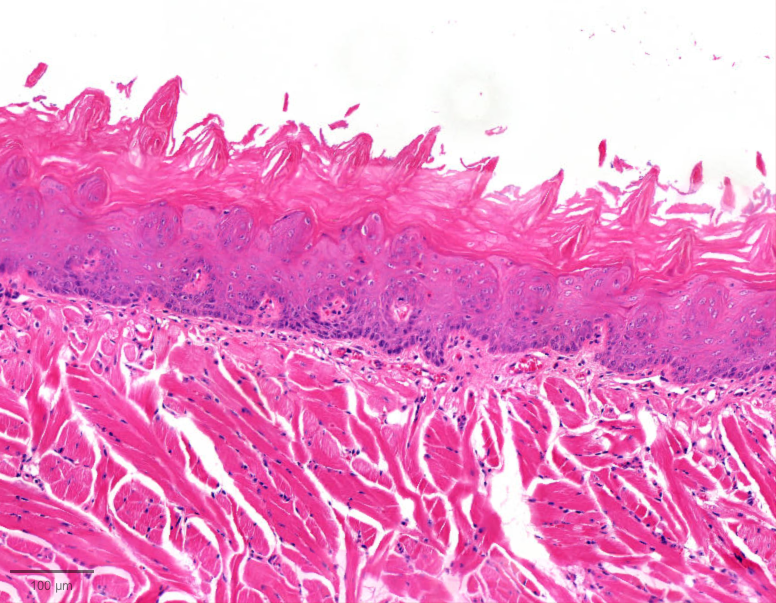

Supplement: Supplementary file 9 — Figure EV6 Source Data [file 44321_2025_256_MOESM9_ESM.zip › Figure EV6-source data/Figure EV6-A/(Revised version)EV6A-OLK-case1-10x.tif]

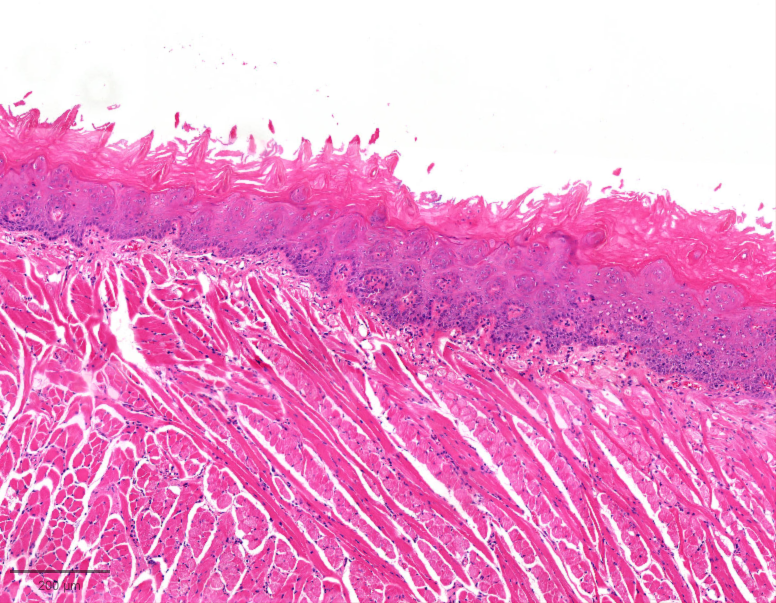

Supplement: Supplementary file 9 — Figure EV6 Source Data [file 44321_2025_256_MOESM9_ESM.zip › Figure EV6-source data/Figure EV6-A/(Revised version)EV6A-OLK-case1-5x.tif]

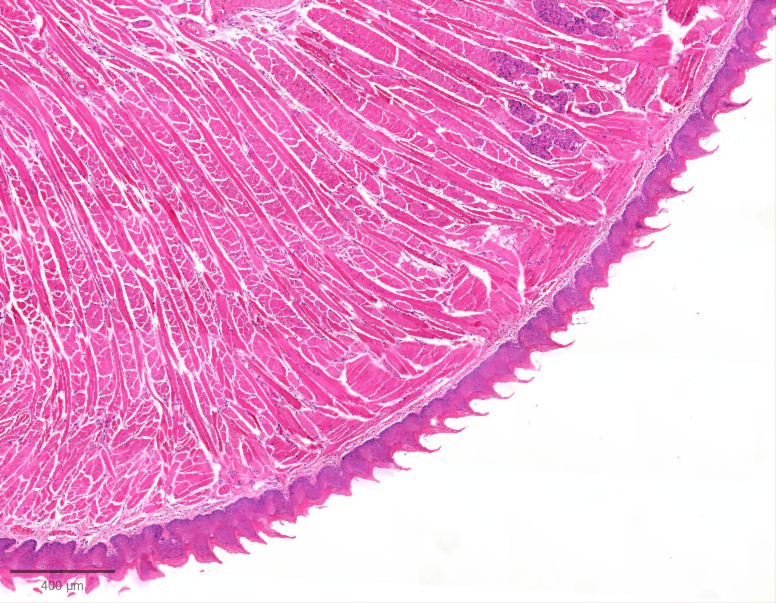

Supplement: Supplementary file 9 — Figure EV6 Source Data [file 44321_2025_256_MOESM9_ESM.zip › Figure EV6-source data/Figure EV6-A/(Revised version)EV6A-Normal-5x.tif]

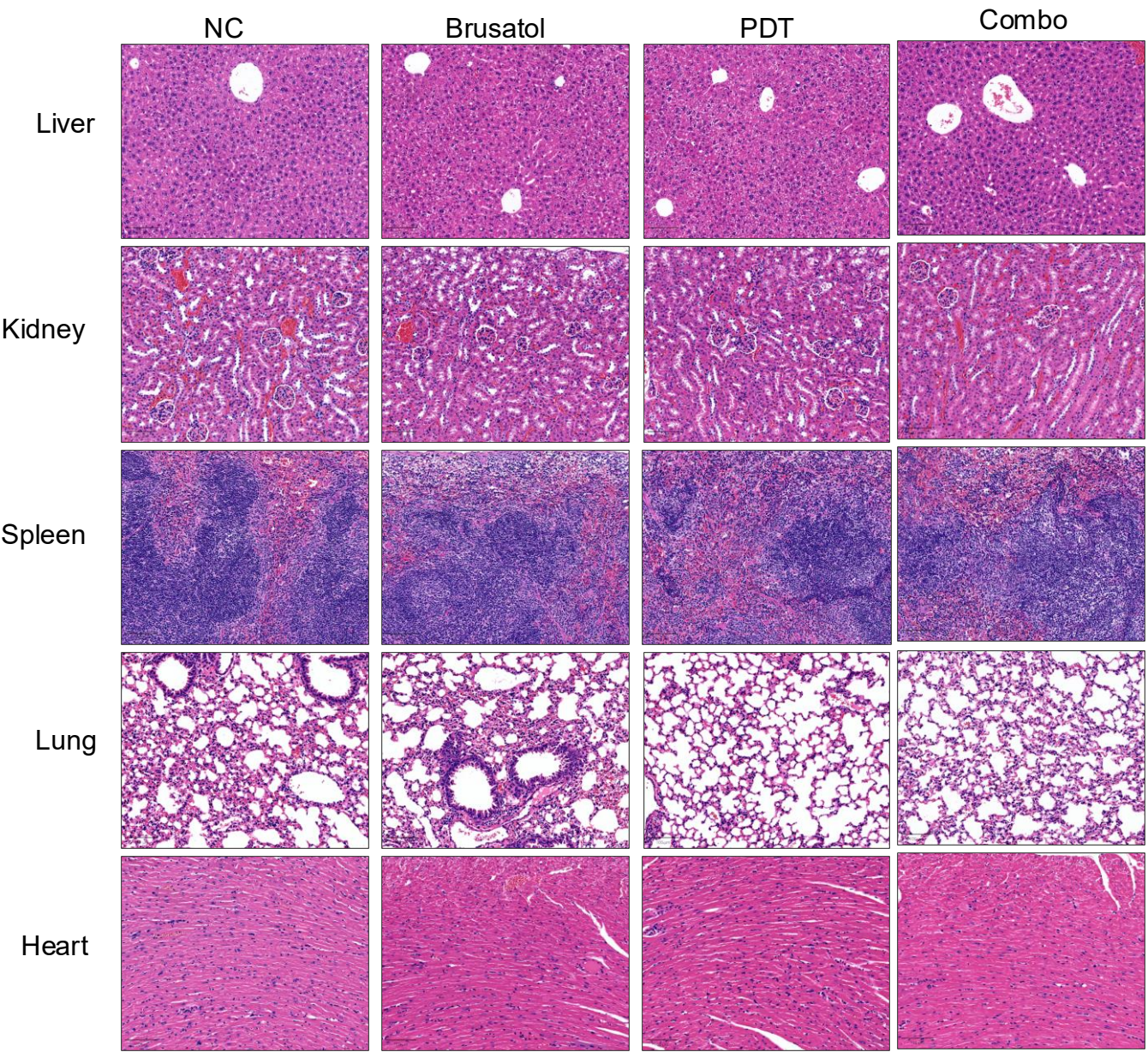

Supplement: Supplementary file 9 — Figure EV6 Source Data [file 44321_2025_256_MOESM9_ESM.zip › Figure EV6-source data/Figure EV6-C/Figure EV6 C.pdf]

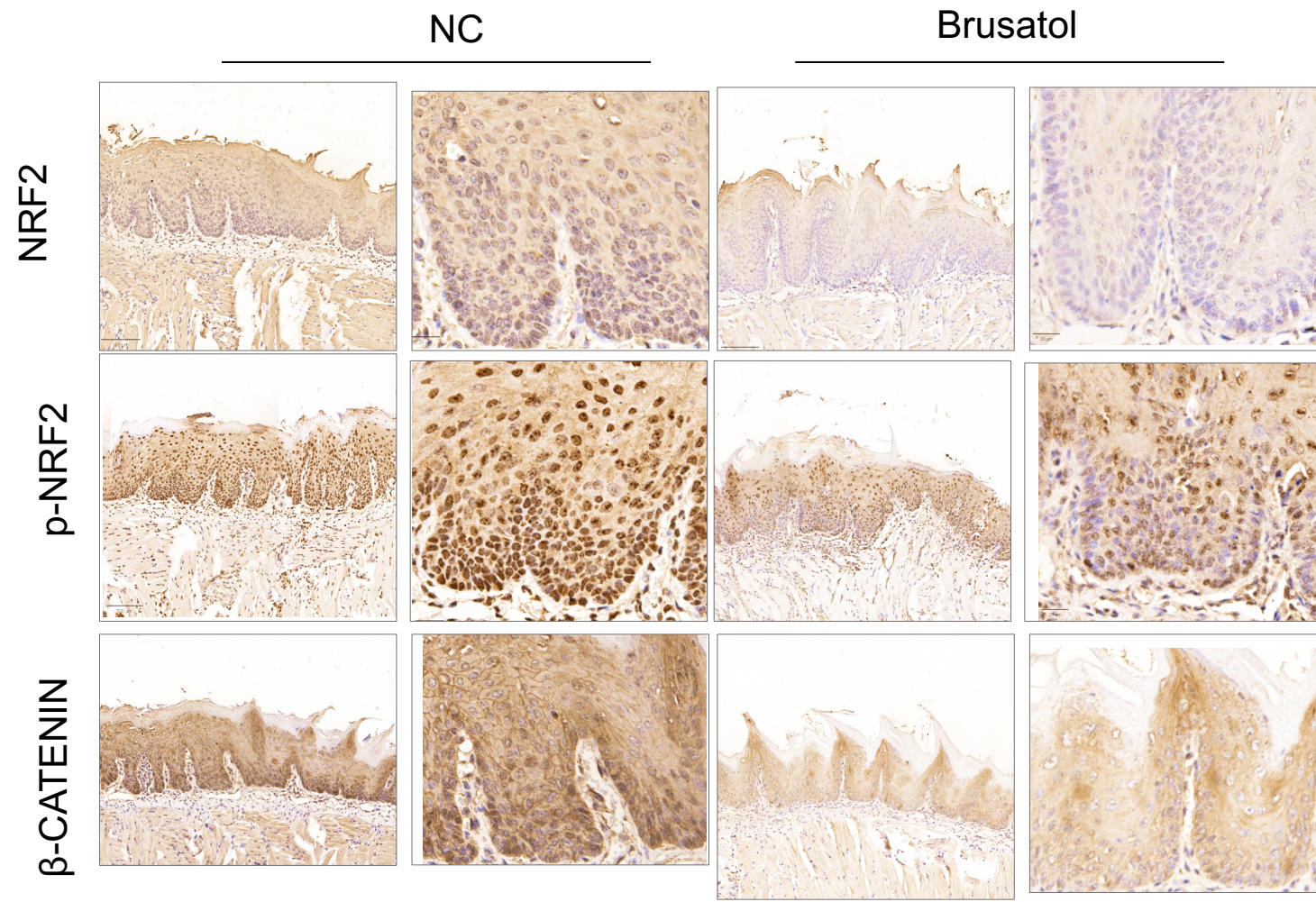

Supplement: Supplementary file 9 — Figure EV6 Source Data [file 44321_2025_256_MOESM9_ESM.zip › Figure EV6-source data/Figure EV6-B/Figure EV6 B (left).pdf]
