# Supplementary material for: NRF2 modulates WNT signaling pathway to enhance photodynamic therapy resistance in oral leukoplakia
Source: EMBO Mol Med. 2025 Jun 10;17(7):1794–824. doi: 10.1038/s44321-025-00256-w (PMC12254380; doi:10.1038/s44321-025-00256-w)
Supplement: Supplementary file 10 — Expanded View Figures [file 44321_2025_256_MOESM10_ESM.pdf]

Expanded View Figures

Figure EV1. The UMAP visualization of cells from six patients with OLK.

(**A**) Schematic diagram illustrating the inclusion and exclusion criteria for sample selection in scRNA-seq. (**B**) Distribution of mitochondrial gene expression percentage (percent_mt) in scRNA-seq. (**C**) Distribution of total RNA counts (nCount_RNA) in scRNA-seq. (**D**) Identification of highly variable genes based on scRNA-seq. Volcano plot showing highly variable genes, with each dot representing a single gene. Red dots indicate the 2,000 selected highly variable genes, among which the top 20 are labeled. Black dots represent genes not selected. (**E**) UMAP plot visualizing cells colored by clusters ($n = 14$ clusters). (**F**) UMAP plot visualizing cells colored by samples ($n = 6$ samples). (**G**) Bar plot displays the proportion of subclusters of epithelial cells in each group.

© The Author(s)

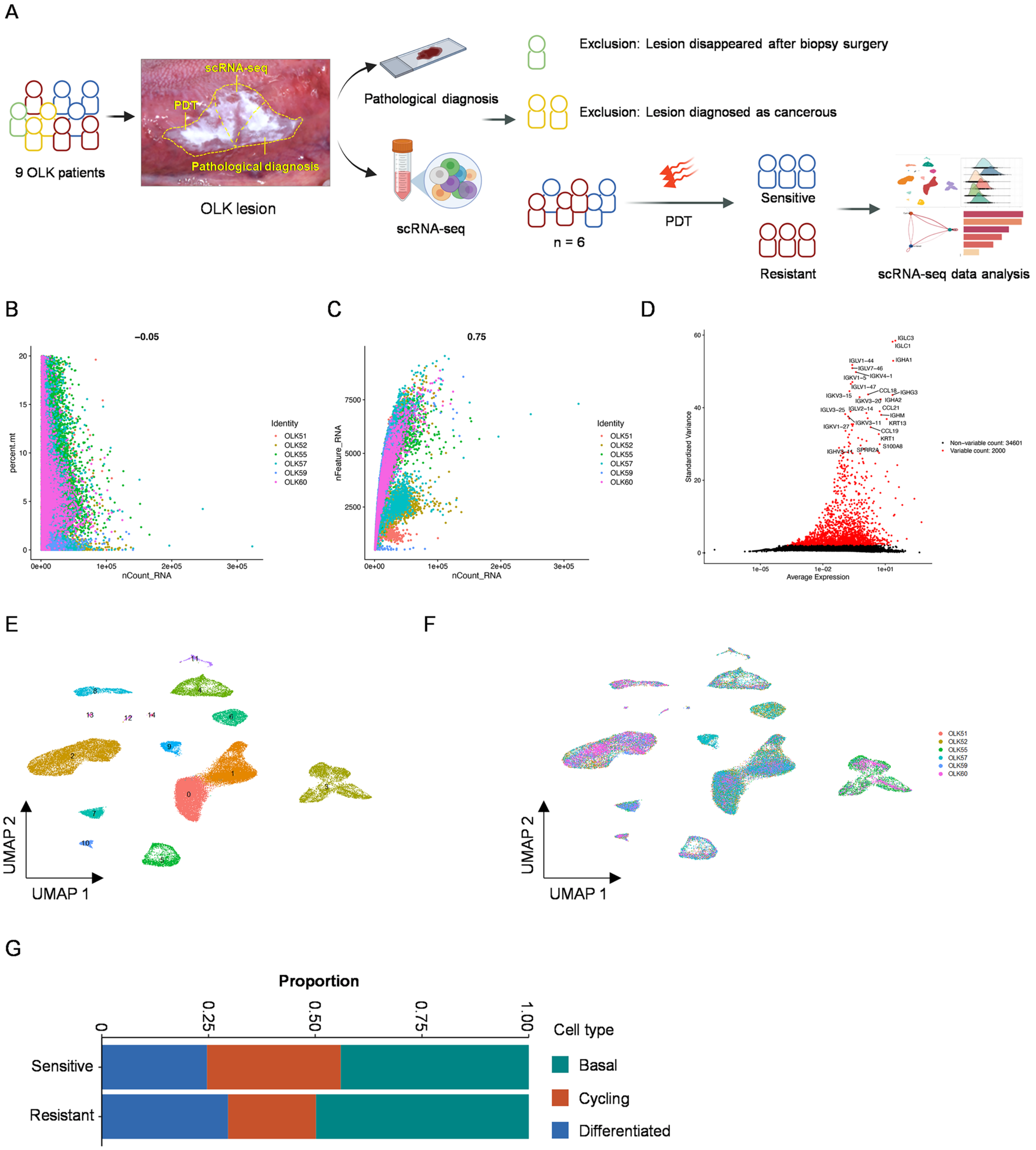
A
9 OLK patients
scRNA-seq
PDT
Pathological diagnosis
OLK lesion
Pathological diagnosis
scRNA-seq
Exclusion: Lesion disappeared after biopsy surgery
Exclusion: Lesion diagnosed as cancerous
n = 6
PDT
Sensitive
Resistant
scRNA-seq data analysis
B
−0.05
percent.mt
nCount_RNA
Identity
OLK51
OLK52
OLK55
OLK57
OLK59
OLK60
C
0.75
nFeature_RNA
nCount_RNA
D
Standardized Variance
Average Expression
Non-variable count: 34601
Variable count: 2000
E
UMAP 2
UMAP 1
F
UMAP 2
UMAP 1
G
Proportion
Sensitive
Resistant
Cell type
Basal
Cycling
Differentiated

 © The Author(s)

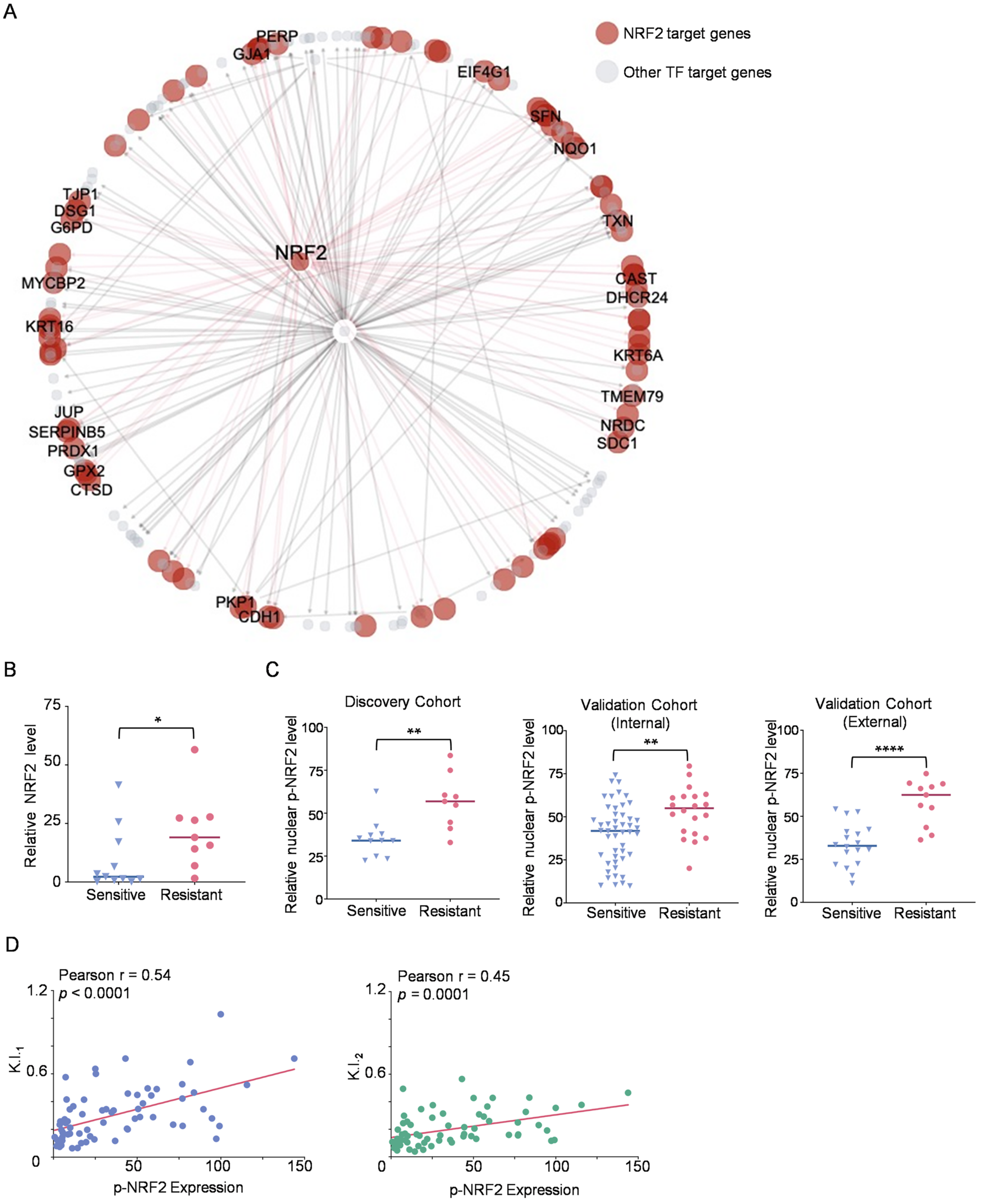
A
NRF2 target genes
Other TF target genes
NRF2
PERP
GJA1
EIF4G1
SFN
NQO1
TXN
CAST
DHCR24
KRT6A
TMEM79
NRDC
SDC1
TJP1
DSG1
G6PD
MYCBP2
KRT16
JUP
SERPINB5
PRDX1
GPX2
CTSD
PKP1
CDH1
B
Relative NRF2 level
Sensitive
Resistant
C
Discovery Cohort
Validation Cohort (Internal)
Validation Cohort (External)
Relative nuclear p-NRF2 level
Sensitive
Resistant
D
Pearson r = 0.54
p < 0.0001
Pearson r = 0.45
p = 0.0001
K.I.1
K.I.2
p-NRF2 Expression

Figure EV2. The expression of NRF2 and p-NRF2 in PDT-resistant OLK patients.

(**A**) Network diagram of NRF2 downstream regulatory target genes. (**B**) Relative NRF2 level in PDT-sensitive ($n = 11$) and resistant ($n = 9$) OLK samples from the discovery cohort in IHC analysis (Student's t-test). (**C**) Relative nuclear p-NRF2 level in PDT-sensitive and resistant OLK samples from the three cohorts in IHC analysis. Sample sizes were: discovery cohort (sensitive: $n = 11$, resistant: $n = 9$), internal validation cohort (sensitive: $n = 48$, resistant: $n = 20$), and external validation cohort (sensitive: $n = 18$, resistant: $n = 11$). Statistical comparisons were performed using Student's t-test. (**D**) Correlation analysis of p-NRF2 expression and tissue K.I. in the internal validation cohort. Pearson correlation significance was assessed using Student's t-test ($n = 68$). Data information: In (**B**–**D**), each data point represents an individual patient (biological replicate). Significance is indicated as *$p < 0.05$, **$p < 0.01$, ****$p < 0.0001$. Exact p-values for these comparisons are provided in Appendix Table S1.

 © The Author(s)

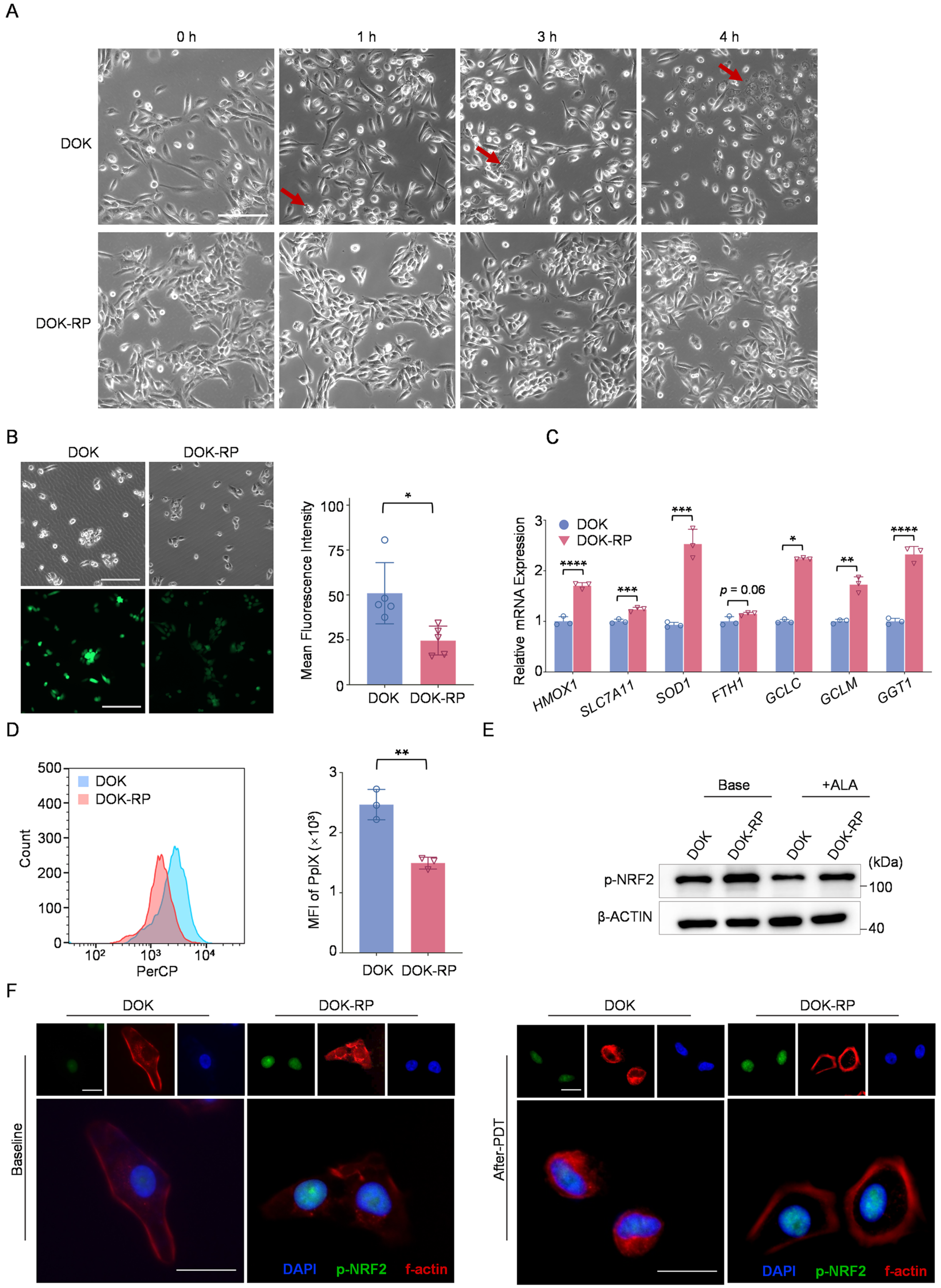
A
0 h
1 h
3 h
4 h
DOK
DOK-RP
B
DOK
DOK-RP
Mean Fluorescence Intensity
100
75
50
25
0
*
DOK
DOK-RP
C
Relative mRNA Expression
DOK
DOK-RP
****
***
***
p = 0.06
*
**
****
HMOX1
SLC7A11
SOD1
FTH1
GCLC
GCLM
GGT1
D
Count
DOK
DOK-RP
PerCP
MFI of PpIX (×10³)
**
DOK
DOK-RP
E
Base
+ALA
DOK
DOK-RP
DOK
DOK-RP
(kDa)
p-NRF2
100
β-ACTIN
40
F
DOK
DOK-RP
DOK
DOK-RP
Baseline
After-PDT
DAPI
p-NRF2
f-actin
DAPI
p-NRF2
f-actin

Figure EV3. Aberrant activation of NRF2 in PDT-resistant OLK cell model.

(**A**) Representative images of morphological changes in DOK and DOK- RP cells after PDT across multiple time points. Scale bar: 50 μm. (**B**) The levels of ROS in PDT-treated DOK and DOK-RP cells were detected using CM-H_2DCFDA probes and observed under fluorescence microscopy. Data represent biological replicates ($n = 5$). Statistical results were presented in the right (Student's t-test). Scale bar: 100 μm. (**C**) Relative mRNA expression of antioxidant stress-related markers in DOK and DOK-RP cells. Data represent biological replicates ($n = 3$). Statistical comparison was performed using Student's t-test. (**D**) Comparison of PpIX uptake by DOK and DOK-RP cells detected by flow cytometry. The statistical results were presented in the right. Data represent biological replicates ($n = 3$). Statistical comparison was performed using Student's t-test. (**E**) Immunoblotting analysis of the expression of p-NRF2 in DOK and DOK-RP cells. (**F**) IF showing the expression of p-NRF2 in DOK and DOK-RP cells. Scale bar: 100 μm. Data information: In (**B**–**D**), data are presented as mean ± SD. Each data point represents an individual biological replicate. Significance is indicated as $^*p < 0.05$, $^{**}p < 0.01$, $^{***}p < 0.001$. Exact *p*-values for these comparisons are provided in Appendix Table S1.

 © The Author(s)

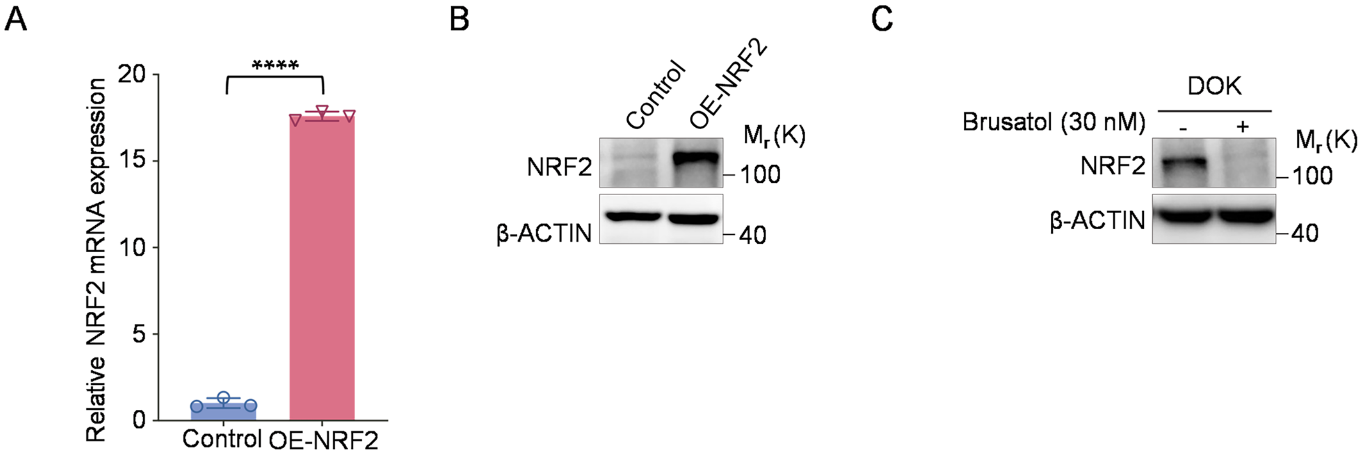

Figure EV4. The expression of NRF2 in OLK cell models.

(**A**) qPCR analyzes relative NRF2 mRNA expression in NRF2 overexpressing DOK cells. Data represent biological replicates ($n = 3$). Statistical comparison was performed using Student's t-test. (**B**) Immunoblotting analyzes the expression of NRF2 in NRF2-overexpressing DOK cell. (**C**) Immunoblotting analyzes the expression levels of NRF2 in DOK cells following Brusatol treatment. Data information: In (**A**), data are presented as mean ± SD. Each data point represents an individual biological replicate. Significance is indicated as ****$p < 0.0001$. Exact p-values for these comparisons are provided in Appendix Table S1.

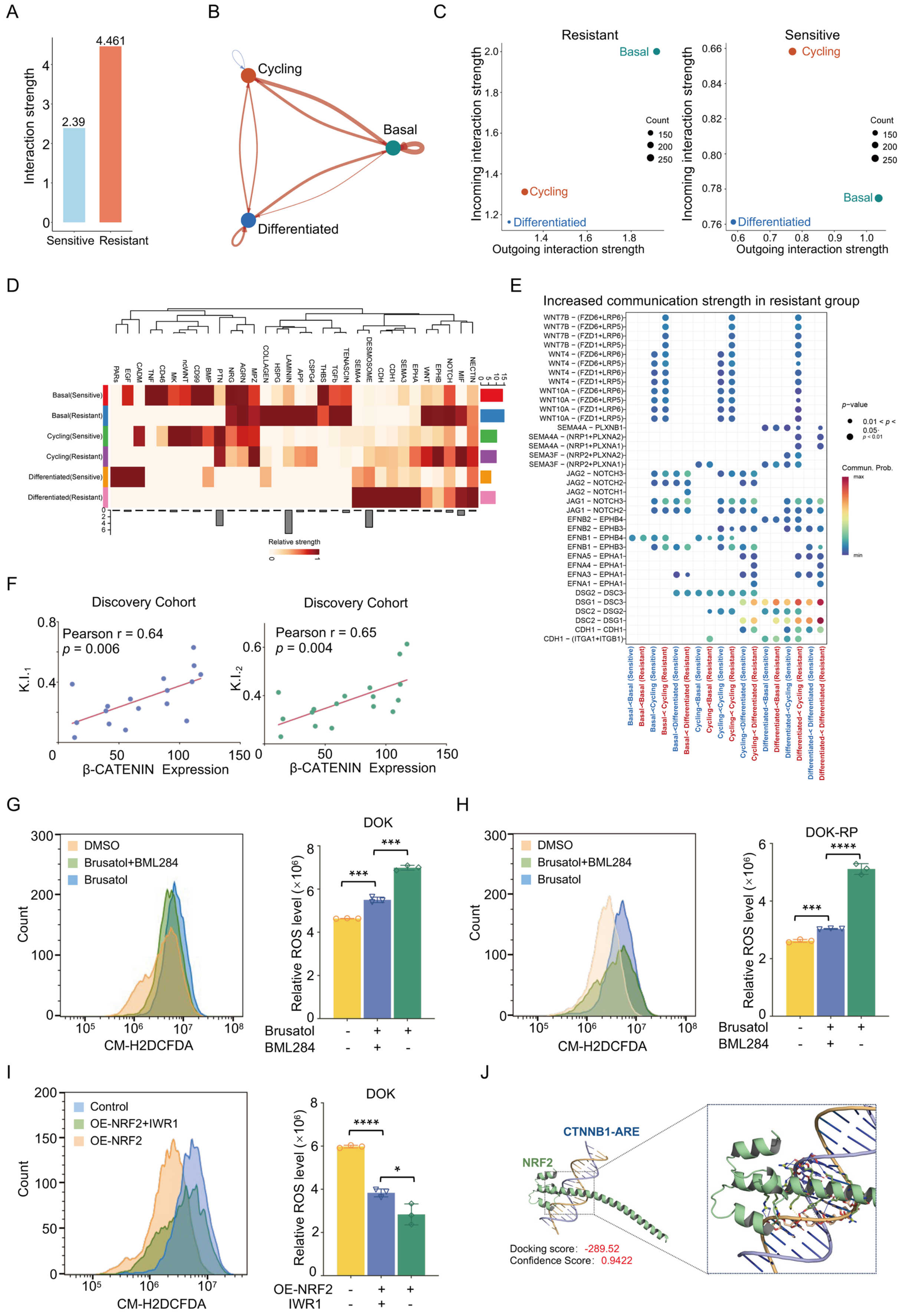
A
Interaction strength
2.39
4.461
Sensitive Resistant
B
Cycling
Basal
Differentiated
C
Resistant
Sensitive
Incoming interaction strength
Outgoing interaction strength
Basal
Cycling
Differentiated
Count
150
200
250
D
Relative strength
E
Increased communication strength in resistant group
p-value
Commun. Prob.
F
Discovery Cohort
Pearson r = 0.64
p = 0.006
Pearson r = 0.65
p = 0.004
β-CATENIN Expression
G
DMSO
Brusatol+BML284
Brusatol
Count
CM-H2DCFDA
DOK
Relative ROS level (×10⁶)
Brusatol
BML284
H
DOK-RP
I
Control
OE-NRF2+IWR1
OE-NRF2
IWR1
J
CTNNB1-ARE
NRF2
Docking score: -289.52
Confidence Score: 0.9422

© The Author(s)

Figure EV5. Contribution of WNT signaling pathway to PDT resistance in OLK cell model.

(**A**) The bar plot showing cellular interaction strength in the sensitive and resistant groups. (**B**) The circle plot displaying the number of receptor/ligand pairs with significant differences between epithelial cell subtypes of the resistant group. (**C**) The bubble plot showing incoming/outgoing interaction strength among subclusters of epithelial cells in each group. (**D**) The heatmap displays intercellular communication signals of various epithelial cell subtypes in the sensitive and resistant groups. (**E**) The bubble plot displays the relative upregulation of ligand receptor signaling among epithelial cell subtypes in the OLK PDT-resistant group. Subpopulations include Basal (sensitive: $n = 1466$ cells; resistant: $n = 799$), Cycling (sensitive: $n = 1042$; resistant: $n = 330$), and Differentiated (sensitive: $n = 820$; resistant: $n = 474$). Statistical analysis was performed using a nonparametric permutation test. (**F**) Correlation between β-CATENIN and tissue K.I. Statistical significance for the Pearson correlation coefficient was assessed using Student's t-test ($n = 17$). (**G**) Flow cytometry showing the effect of combined treatment with BML284 and Brusatol on ROS levels in DOK cells after PDT treatment. Data represent biological replicates ($n = 3$). Statistical comparison was performed using Student's t-test. (**H**) Flow cytometry showing the effect of combined treatment with BML284 and Brusatol on ROS levels in DOK-RP cells after PDT treatment. Data represent biological replicates ($n = 3$). Statistical comparison was performed using Student's t-test. (**I**) Flow cytometry showing the effect of IWR1 on ROS levels in DOK cells overexpressing NRF2 after PDT treatment. Data represent biological replicates ($n = 3$). Statistical comparison was performed using Student's t-test. (**J**) Using HDOCK to predict the binding sites between NRF2 (green) and the ARE sequence (yellow and purple interactions) in the *CTNNB1* promoter. Data information: In (**G–I**), data are presented as mean ± SD. In (**F–I**), each data point represents an individual biological replicate. Significance is indicated as $^*p < 0.05$, $^{***}p < 0.001$, $^{****}p < 0.0001$. Exact *p*-values for these comparisons are provided in Appendix Table S1.

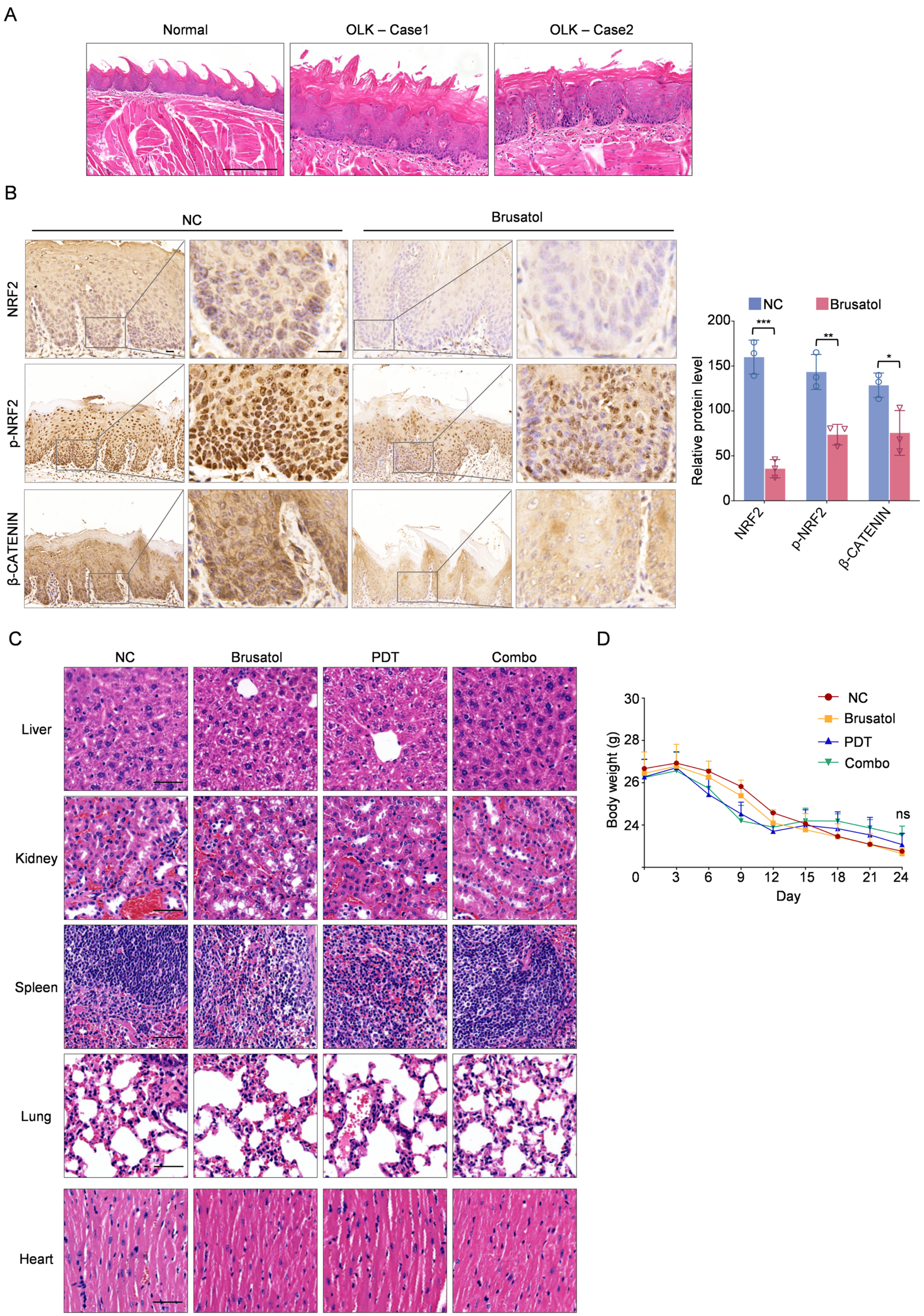
A
Normal
OLK – Case1
OLK – Case2
B
NC
Brusatol
NRF2
p-NRF2
β-CATENIN
NC
Brusatol
Relative protein level
200
150
100
50
0
***
**
*
NRF2
p-NRF2
β-CATENIN
C
NC
Brusatol
PDT
Combo
Liver
Kidney
Spleen
Lung
Heart
D
Body weight (g)
30
28
26
24
0
NC
Brusatol
PDT
Combo
ns
3
6
9
12
15
18
21
24
Day

 © The Author(s)

Figure EV6. Brusatol inhibited the expression of NRF2 and β-CATENIN in the OLK mouse model.

(**A**) Representative HE images of tongue lesions from 4NQO-induced OLK mouse models at week 16 and normal tongue tissues from control mice. Scale bar: 200 µm. (**B**) Representative IHC images of NRF2, p-NRF2, and β-CATENIN expression in tongue tissues from Brusatol-treated and NC mice at day 3 (left, scale bar: 20 µm). Each group included $n = 3$ mice. Relative IHC expression levels of NRF2, p-NRF2, and β-CATENIN were quantified and compared between groups (right). Statistical comparison was performed using Student's t-test. (**C**) Representative HE images of the liver, kidney, spleen, lung, and heart from mice in each group. Scale bar: 50 µm. (**D**) Weight changes of mice in each group before and after treatment ($n = 4$ mice per group). Statistical comparison was performed using one-way ANOVA followed by Tukey's post hoc test. Data information: In (**B**, **D**), data are presented as mean ± SD. Each data point represents an individual biological replicate. Significance is indicated as *$p < 0.05$, **$p < 0.01$, ***$p < 0.001$, ns: not significant ($p \geq 0.05$). Exact p-values for these comparisons are provided in Appendix Table S1. Source data are available online for this figure.

© The Author(s)
