# Supplementary material for: NRF2 modulates WNT signaling pathway to enhance photodynamic therapy resistance in oral leukoplakia
Source: EMBO Mol Med. 2025 Jun 10;17(7):1794–824. doi: 10.1038/s44321-025-00256-w (PMC12254380; doi:10.1038/s44321-025-00256-w)

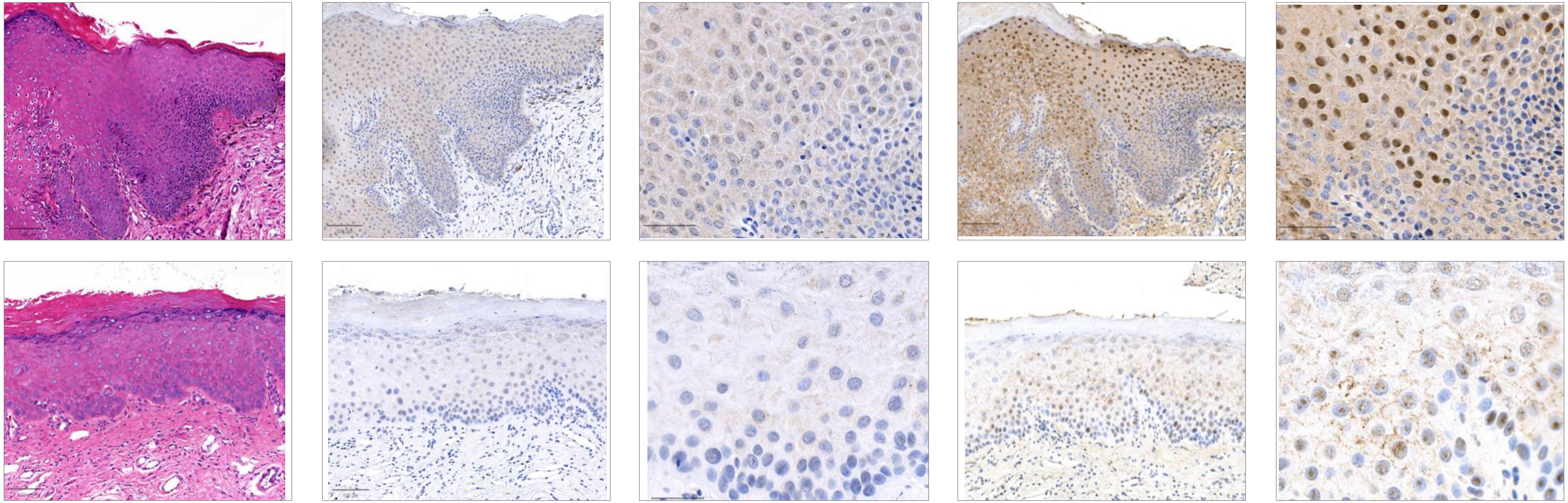

Supplement: Supplementary file 4 — Source data Fig. 2 [file 44321_2025_256_MOESM4_ESM.zip › Figure 2-source data/2E/Figure 2E.pdf.zip!/Figure 2E.pdf]
