# Supplementary material for: NRF2 modulates WNT signaling pathway to enhance photodynamic therapy resistance in oral leukoplakia
Source: EMBO Mol Med. 2025 Jun 10;17(7):1794–824. doi: 10.1038/s44321-025-00256-w (PMC12254380; doi:10.1038/s44321-025-00256-w)

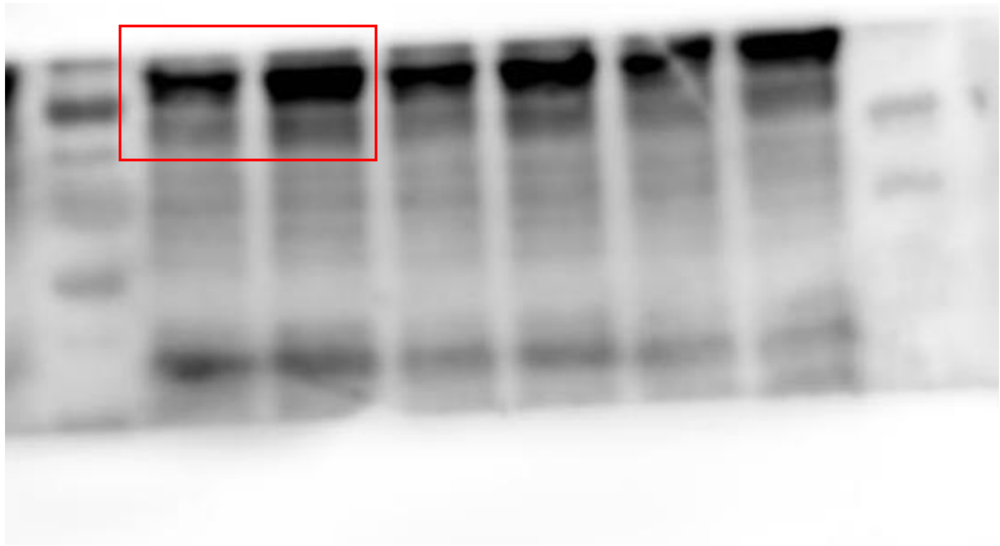

Supplement: Supplementary file 5 — Source data Fig. 3 [file 44321_2025_256_MOESM5_ESM.zip › Figure 3-source data/Figure 3I/Figure 3I.zip!/LOR.pdf]
