# Supplementary material for: NRF2 modulates WNT signaling pathway to enhance photodynamic therapy resistance in oral leukoplakia
Source: EMBO Mol Med. 2025 Jun 10;17(7):1794–824. doi: 10.1038/s44321-025-00256-w (PMC12254380; doi:10.1038/s44321-025-00256-w)
Supplement: Supplementary file 6 — Source data Fig. 4 [file 44321_2025_256_MOESM6_ESM.zip › Figure 4-source data/Figure 4C/Figure 4C.pdf.zip!/Figure 4C.pdf]

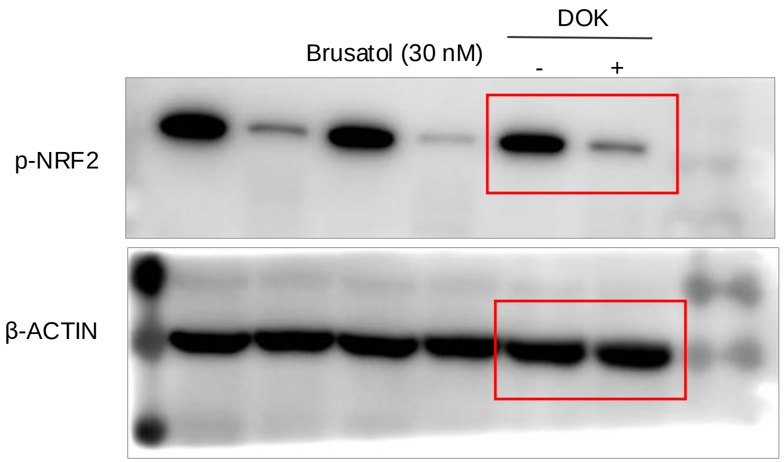
DOK
Brusatol (30 nM)
-
+
p-NRF2
β-ACTIN

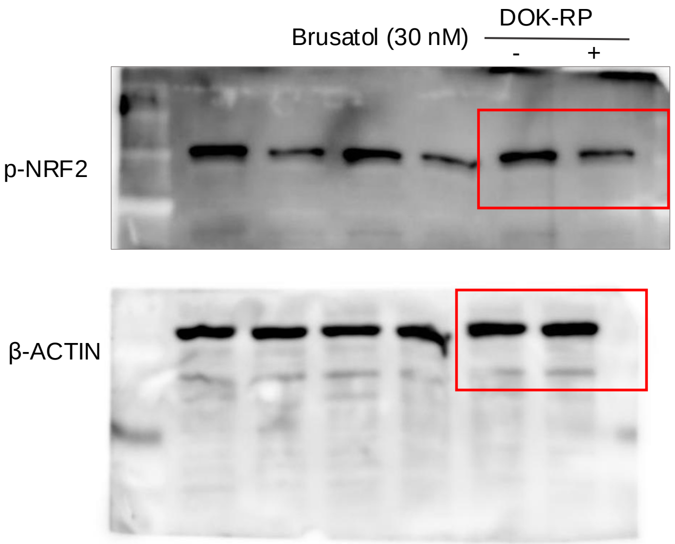
DOK-RP
Brusatol (30 nM)
-
+
p-NRF2
β-ACTIN
